# Modeling Peptide Nucleic Acid Binding Enthalpies Using MM-GBSA

**DOI:** 10.1021/acs.jpcb.2c05547

**Published:** 2022-11-14

**Authors:** Jack Goodman, David Attwood, Janice Kiely, Pablo Coladas Mato, Richard Luxton

**Affiliations:** † University of the West of England, BristolBS16 1QY, U.K.; ‡ GKN Aerospace, BristolBS34 6FB, U.K.

## Abstract

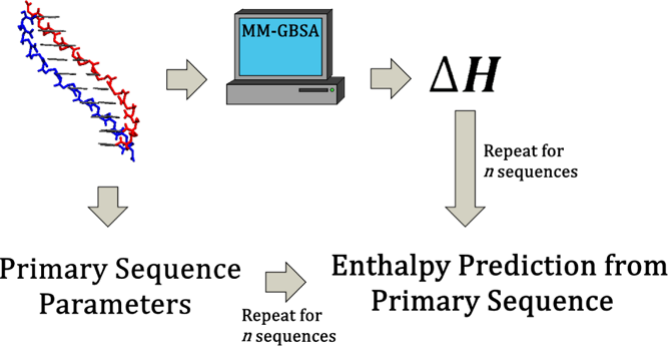

The binding enthalpies of peptide nucleic acid (PNA) homoduplexes were predicted using a molecular mechanics generalized Born surface area approach. Using the nucleic acid nearest-neighbor model, these were decomposed into sequence parameters which could replicate the enthalpies from thermal melting experiments with a mean error of 8.7%. These results present the first systematic computational investigation into the relationship between sequence and binding energy for PNA homoduplexes and identified a stabilizing helix initiation enthalpy not observed for nucleic acids with phosphoribose backbones.

## Introduction

**Figure 1:**
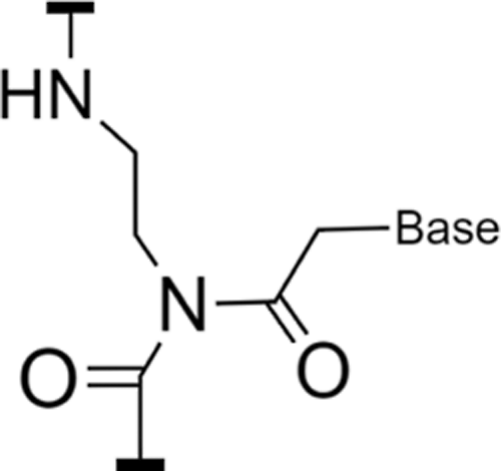
Schematic of an *N*-(2-aminoethyl)glycine monomer of a PNA backbone connected to a base through a carbonyl methylene linker.

The binding of nucleic acids is a sequence-dependent process facilitated primarily through the Watson–Crick pairing of complementary base pairs. This is not a property unique to naturally occurring nucleic acids such as DNA or RNA. It is therefore possible to produce nucleic acid analogue molecules which adhere in a sequence-specific manner but with modified properties. Peptide nucleic acid (PNA) is an analogue wherein the phosphoribose backbone of DNA or RNA has been entirely replaced. Instead, PNA has a backbone composed of repeating *N*-(2-aminoethyl)glycine monomers (Figure 1).^1^ The directionality of the backbone proceeds from an N-terminal amine to a C-terminal carboxyl, similar to a polypeptide, and PNA forms both stable homo- and heteroduplex structures in solution.^2,3^

**Figure 2:**
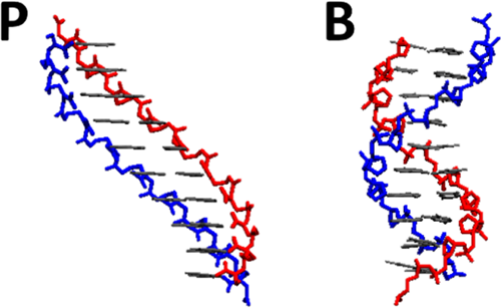
10 base-pair structures of P-form PNA (left) and B-form DNA (right).

PNA homoduplexes form P-form^1,3^ double helices in solution. The P-form helix has a large 18 base-pair pitch and a wide 28 Å helical diameter (Figure 2). PNA/DNA heteroduplexes have a structure intermediate of the P- and B-forms, demonstrating that PNA is flexible enough to remain stable in its non-preferred helical conformation.^4^

Despite adopting non-preferred helical structures, PNA heteroduplexes are more thermally stable than DNA or RNA homoduplexes, though they are less stable than PNA homoduplexes.^3^ Unlike DNA or RNA homoduplexes, PNA heteroduplex stability is largely independent of the concentration of salt in solution.^2,5^ This is due to the PNA neutral backbone since the net repulsion between the negatively charged phosphoribose backbones in DNA or RNA is not present.^6^ As a result, the PNA backbone does not need to associate with counterions to stabilize the double helix.^2^

PNA heteroduplexes are also biostable since they are not substrates for nucleases and proteases.^5^ The acidic conditions that would depurinate DNA or RNA homoduplexes also typically do not depurinate PNA.^7^ Since PNA remains stable under a variety of conditions, it has attracted interest for applications ranging from antisense and antigene technologies to biosensing, genetic diagnostics, molecular biology, and medicinal chemistry. For example, PNA has been applied in vivo to silence gene transcription and translation.^8−10^

The PNA backbone can be modified to produce favorable properties in heteroduplexes. For example, the incorporation of lysine or arginine functional groups at the α carbon of the backbone increased the electrostatic forces of attraction between a PNA strand and a DNA complement.^11^ Similar modifications could be applied to PNA homoduplexes, with the advantage that identical chemistry can be used for both strands. Given the higher thermal and environmental stabilities of PNA relative to other nucleic acids with biotechnological applications, it is feasible that PNA technologies based on such modifications could have desirable properties.

PNA homoduplex technologies, however, must be preceded by a deeper understanding of the relation between the PNA structure and binding properties. Currently, no model exists that decomposes the binding energies of PNA duplexes into sequence parameters. All-atom molecular dynamics is an appropriate means to develop such a model since any number of PNA structures can be easily generated, and the expense of specialist equipment such as synthesizers can be avoided.

To this end, molecular mechanics generalized Born surface area (MM-GBSA) analysis of simulated PNA structures was conducted. Entropy was estimated using a quasi-harmonic (QH) approximation. The current force field parameters designed by Jasiński et al.^12^ were implemented. The computational results were benchmarked against thermal melting data obtained both from this work and the available literature.

Using a previously developed method,^13^ it was determined that nearest-neighbor decomposition of PNA binding enthalpies was possible. Entropy and free energy could also be obtained from experiments, though benchmarking the QH entropy revealed that it was a poorer estimate. Finally, it was found that sequence parameters for binding enthalpies could be understood in terms of the dynamics of PNA homoduplexes through hydrogen bonding analysis.

## Theory and Methods

### Literature Analysis

Seven PNA homoduplexes with binding energies and entropies from thermal melting experiments were compiled from the literature.^2,3,14−18^

Experimental conditions used by the publications were inconsistent. Namely, the concentration of sodium ions and the use of a solubility-enhancing lysine tag were varied. Since prior research consistently agreed that PNA stability is negligibly affected^2,5,6^ by a change in the concentration of sodium chloride, this was not considered critical.

To determine that lysine tagging did not affect the binding energies, the sequence GTAGATCACT, which appeared the most times in the published research, was categorized according to whether this tag was present or not. It was found that the binding energies and entropies for unmodified sequences were all within one standard error of the mean binding energies and entropies for sequences with the tag (Table S1).

Sequences were therefore grouped into a single data set regardless of these conditions.

### Thermal Melting Experiments

Three PNA homoduplexes, CGATCG, AACGTT, and TAGCTA, were purchased from Eurogentec at 95% purity (Cat. Number BA-PN010-005) as determined by high-performance reverse-phase and ion-exchange liquid chromatography methods. Samples were diluted to a concentration of 1 μM in deionized water and were then melted via an electric heater in a water bath from which samples were taken at 2 K intervals after 5 min equilibrations at the target temperature. The absorbances of the PNA strands at 260 nm were then monitored using an Agilent Technologies Cary 60 UV–vis spectrophotometer. Their absorbances increased during melting because single-stranded nucleic acids are hyperchromic at 260 nm relative to the duplex.

Absorbances were normalized between zero and one to produce alpha curves, where α = 1 corresponds to a sample containing only double helices, while α = 0 contains only single strands. The melting point was defined at α = 0.5. The equilibrium constant was then derived according to a two-state assumption for the transition using the equation for self-complementary helices.^19^
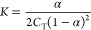
1where *C*_T_ is the total strand concentration. The natural logarithm of the equilibrium constant was then plotted against the reciprocal temperature to derive the standard enthalpies and entropies of the transition according to the linear form of the van’t Hoff equation. This assumes that the entropy and enthalpy are temperature-independent.
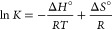
2

The free energy of binding of the strands was then calculated according to Δ*G* = Δ*H* – *T*Δ*S*.

### Molecular Dynamics

Simulated structures of PNA homoduplexes were generated using the proto-Nucleic Acid Builder .^20^ A set of initial helical parameters^21−23^ were obtained from published crystal or NMR structures. A weighted Monte Carlo conformational search was conducted to generate chemical structures based on these initial parameters and on thresholds such as bonding energies and interatomic distances set by the user. The structures generated by this were imported into the Gromacs program using the July 2021 edition of the CHARMM36m forcefield,^24,25^ and the N- and C-termini were acetylated and amidated, respectively.

Simulated homoduplexes were energy minimized in vacuum over 50,000 simulation steps using a steepest-descent algorithm. Structures were then solvated in explicit waters in dodecahedral boxes using the simple point charge (SPC) water model spc216.^26^ The distance between any atom of the solute and the edge of the box was set to a minimum of 15 Å. Periodic boundary conditions^27^ were enforced to imitate a bulk solvent, and solvated structures were energy minimized using steepest-descent minimization over 50,000 simulation steps.

For each PNA homoduplex, three replicates were obtained from this point onward. Each replicate was heated from 30 to 298 K over 500 ps, and Newton’s equations of motion were integrated using a leapfrog integrator.^28,29^ Temperature was weakly coupled to an external bath using a velocity rescaling^30^ algorithm. Harmonic restraints of 24 kcal mol^–1^ Å^–1^ were applied to non-hydrogen atoms, whereas hydrogen atoms were constrained using the LINCS^31^ algorithm for the suppression of high-frequency hydrogen oscillations such that 2 fs timesteps could be used. During this and for all future simulations, the van der Waals interactions were handled using a switched cut-off scheme, with switching to zero occurring from 10 to 12 Å. The electrostatic nonbonded interactions were treated using a particle mesh Ewald (PME)^32,33^ algorithm with quartic interpolation and a grid spacing of 1 Å. The short-ranged component of the PME was computed to 10 Å, and long-ranged components were handled using the fast Fourier transform library FFTW.^34^

After heating, position restraints were halved every 2 ns, with the equations of motion and temperature coupling thereon handled by a stochastic integrator, which incorporated friction and noise terms.^35^ After 10 ns of simulation, the non-hydrogen restraints were removed entirely, and structures were equilibrated for 10 ns in the canonical ensemble. They were then equilibrated for a further 10 ns in the isobaric–isothermal ensemble with pressure coupling at 1 bar handled by a Berendsen barostat.^36^ 100 ns production runs were then carried out for each of the three replicas of each sequence using Parrinello–Rahman pressure coupling.^37^

### Trajectory Analysis

Trajectories were analyzed by gmx_mmpbsa, which enables MM-GBSA estimations of enthalpy, entropy, and free energy to be calculated.^38^ The free energy of a molecule computed using MM-GBSA^39−41^ is given by the average molecular mechanics potential energy *E*_MM_, the solvation energy *G*_solv,_ and the entropic term TS. The difference in free energies of two single PNA strands and the PNA homoduplex is then given by the difference of these.
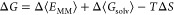
3*E*_MM_ is the sum of the bonded and nonbonded interactions of the molecule in vacuum, obtained from snapshots of the simulation trajectory, and accounts for the bond angle and dihedral energies between bonded atoms and the van der Waals and electrostatic interactions between nonbonded atoms.
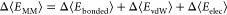
4

In the single-trajectory approach used in this project, the individual PNA strands are isolated from the trajectory of the homoduplex, meaning that . The single-trajectory approach assumes that the conformation of a PNA strand in the duplex is similar to a free PNA strand in solution but is computationally less expensive. The validity of the approach was tested by simulating three single-stranded PNAs, for a total of nine replicates, and calculating their enthalpies using the same generalized Born method. These enthalpies were then compared to the enthalpies of the single strands as evaluated by the single-trajectory approach, that is, when taken from the trajectory of the duplex. The difference in this conformation enthalpy^42,43^ was limited (∼1%) in all three cases (Table S2).

The solvation energy difference  from eq 3 is calculated using a continuum solvent model, which approximates the explicit water solvent as a homogeneous medium with a dielectric constant of 78.5 at 298 K. It is composed of polar and nonpolar components. The polar term accounts for the electrostatic interactions between solute and solvent. The nonpolar component accounts for the work of formation of a cavity the size of the solute in the solvent and the short-ranged van der Waals interactions between the solute and the solvent.^44^
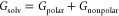
5a
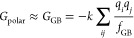
5b
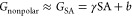
5cwhere *q*_*i*_ and *q*_*j*_ are atomic charges and *k*, in the generalized Born model, is a function of the dielectric constants of the solvent and of free space, where it is unity. *f*_GB_ is a function of interatomic distances and Born radii of the *i*th and *j*th atoms, wherein the Born radius is the spherically averaged distance between an atom of the solute and the solvent. *G*_SA_ is a linear function of the solute’s solvent-accessible surface area (SA).^45^

The final contribution to the free energy change according to eq 4 is then *T*Δ*S,* which was evaluated in this study using a QH approximation.^46^

Using the MM-GBSA approach, the binding energies for PNA homoduplexes were evaluated. Snapshots from each production trajectory were stripped of explicit water, and the first 20 ns were discarded. The remaining 80 ns were analyzed using MM-GBSA. The version of the generalized Born model*,igb* = 5,^47^ was used for the analysis, and enthalpy change was taken to be  according to Δ*G* = Δ*H* – *T*Δ*S*.

A standard state correction was applied to the free energy differences to correct for the box volumes and thereby standardize the concentrations.
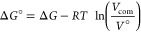
6where *V*° = 1661 Å^3^ and *V*_com_ is the volume of the simulation box.^48^

The production trajectories with explicit waters were then analyzed using gmx_rms and gmx_hbond, which were used to compute the root-mean-square atomic deviations (rmsds) (Figure S1) over time and the number of Watson–Crick hydrogen bonds between the base pairs over time. Hydrogen bonds were defined as bonds between the atom triplets involved in Watson–Crick bonding with distances between donor and acceptor of less than 0.325 nm and angles of hydrogen-donor–acceptor of less than 30°.

To confirm that run durations were sufficient to obtain converged thermodynamic data, relative enthalpies, entropies, and free energies were plotted as functions of sequence length (Figures S2–S4). To determine whether simulations were equilibrated and repeatable, rmsd histogram distributions for the replicas of each sequence were overlapped (Figures S5 and S6).

### Modeling Nucleic Acid Binding Energies

**Figure 3:**
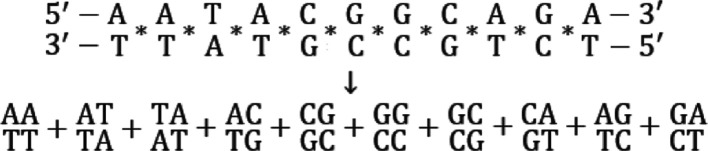
An oligonucleotide duplex broken down into a series of overlapping stacks, as indicated by the asterisks. Stacks are written below the duplex, as indicated by the arrow.

A nucleic acid duplex can be thought of as a series of overlapping base pair stacks (Figure 3). A stack is two base pairs in sequence, and there are 10 unique stacks for homoduplexes. Overlapping means that the first pair of the following stack is the second pair of the preceding stack. This is known as the nearest-neighbor model. Given that the stabilities of nucleic acid duplexes are impacted both by their base-pairing and base-stacking energies, the nearest-neighbor method is theoretically rigorous in that it accounts for both.^49−51^

The energies and entropies of binding are given by summing over the energies and entropies of these stacks. In addition, the helix initiation energy or entropy and the terminal guanine–cytosine (GC) energy or entropy are added. The binding energy or entropy of a sequence is then a sum over the stacks, the helix initiation term, and the terminal GC term.
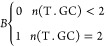
7a
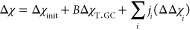
7bwhere Δχ is a binding energy or entropy. Δχ_init_ is a helix initiation energy or entropy. Δχ_T.GC_ is the energy or entropy added so long as the number of terminal GC base pairs, *n*(T.GC), is two, as indicated by eq 7a. Put differently, *B*Δχ_T.GC_ evaluates to zero if there is a terminal adenine-thymine (AT) base pair. ΔΔχ_*i*_ is the incremental change in binding energy or entropy given by the *i*th unique base pair with occurrence *j*_*i*_.

For self-complementary helices, the binding entropy is decreased by 1.4 cal K^–1^ mol^–1^ to account for the *C*2 rotational symmetry of the double helix, which reduces the degrees of freedom upon binding.^51^

Equation 7b has the matrix form
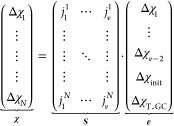
8where **χ** is a 1 × *N* column matrix containing either mean enthalpies, entropies, or free energies of hybridization of PNA homoduplexes. ***S*** is an *N* × *v* matrix containing the occurrences *j* of all stacked base pairs, terminal GC and initiation terms. **ε** is a 1 × *v* column matrix containing the unknown energies or entropies of these terms. These unknowns are then evaluated by solving the linear least-squares problem, which minimizes the sum of the square of the residuals of **χ** with respect to ***S***.

The problem is generalized by weighting each observable Δχ by its reciprocal standard error.
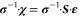
9where **σ**^–1^ is an *N* × *N* diagonal matrix containing the reciprocal standard errors of each observable with non-diagonal elements of zero. The estimate **ε** obtained by solving the multiple linear regression problem thus contains the error-weighted least-squares estimates of the energies or entropies of the terms on the right-hand side of eq 7b.

Equation 9 can be used to decompose MM-GBSA estimates of binding enthalpies, entropies or free energies for PNA homoduplexes into sequence parameters.

## Results and Discussion

### Thermal Melting Data

**Figure 4:**
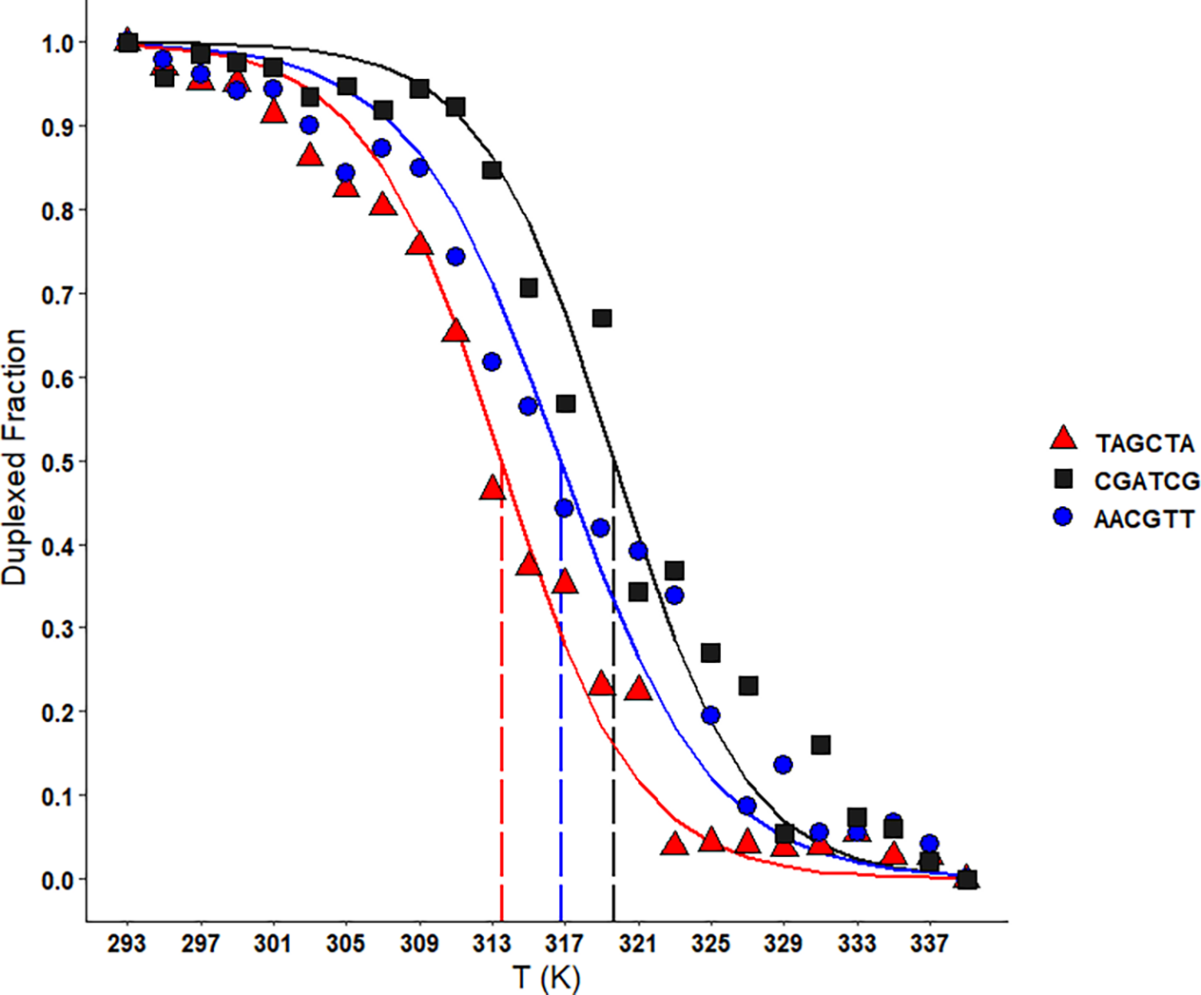
Normalized melting curves for the three PNA hexamers TAGCTA, CGATCG, and AACGTT, as measured by UV melting experiments. The duplexed fraction, α, is indicated on the *y* axis and the temperature in kelvin on the *x* axis. Melting points are shown by the dashed vertical lines. Points represent the means of independent triplicates and were obtained at 2 K intervals.

Melting points of the three PNA hexamers defined as α = 0.5 were observed at 313.5 ± 0.4, 316.8 ± 0.4, and 319.7 ± 0.8 K for TAGCTA, AACGTT, and CGATCG, respectively (Figure 4).

Using eqs 1 and 2, the standard binding free energies, enthalpies and entropies were determined for the three hexamers. The standard errors of the measured enthalpies and entropies for all three sequences overlapped, whereas the free energy of hybridization for CGATCG was approximately 1 kcal mol^–1^ more negative. This suggested that CGATCG was stabilized relative to the other two homoduplexes as was corroborated by their melting temperatures.

The binding energies and entropies of the three sequences were compiled alongside the binding energies and entropies obtained from the literature. This produced a data set totaling 10 sequences, 7 of which were obtained from the literature,^2,3,14−18^ against which computationally derived energies and entropies could be benchmarked (Table 1).

**Table 1:** Standard Free Energies, Enthalpies, and Entropies of Binding for the 10 PNA Homoduplexes from Thermal Melting Experiments^a^

sequence	–Δ*G*_298_^o^(kcal mol^–1^)	–Δ*H*_298_^o^(kcal mol^–1^)	–Δ*S*_298_^o^(cal K^–1^ mol^–1^
(a) Experimental Thermal Melting Data			
CGATCG	13.64 ± 0.25	58.78 ± 4.09	151.42 ± 12.89
AACGTT	12.64 ± 0.39	52.05 ± 5.30	132.24 ± 16.45
TAGCTA	12.34 ± 0.29	55.08 ± 3.85	143.41 ± 11.95
(b) Literature Thermal Melting Data			
GTAGATCACT^b^^,^^c^^,^^d^^,^^e^^,^^f^^,^^g^	18.99 ± 0.87	86.92 ± 5.00	227.97 ± 14.48
TGTTACGACT^h^	21.08 ± 1.00	92.60 ± 5.70	240.00 ± 16.11
AGGTAACCAG^d^	18.76 ± 0.60	83.30 ± 2.80	216.60 ± 7.40
AGTGAAGCAG^d^	19.02 ± 0.82	82.15 ± 4.56	211.85 ± 12.86
TGATCTAC^f^	13.10 ± 0.00	60.90 ± 0.00	224.70 ± 0.00
GTAGATCACTGT^f^	21.40 ± 0.00	97.10 ± 0.00	253.80 ± 0.00
GTAGATCACTGTCAC^f^	26.40 ± 0.00	117.10 ± 0.00	304.30 ± 0.00

a Standard errors are as provided by the study author or are the standard errors from reported energies or entropies of multiple publications if a sequence is reported multiple times. Standard errors of zero indicate that they were not standard errors but provided by the study author.

b Tomac, S.; Sarkar, M.; Ratilainen, T.; Wittung, P.; Nielsen, P.; Nordén, B.; Gräslund, A. Ionic Effects on the Stability and Conformation of Peptide Nucleic Acid Complexes. *J. Am. Chem. Soc*. **1996,***118,* 5544–5552.

c Sen, A.; Nielsen, P. On the stability of peptide nucleic acid duplexes in the presence of organic solvents. *Nuc. Acid. Res*. **2007,***35,* 3367–3374.

d Sen, A.; Nielsen, P. Unique Properties of Purine/Pyrimidine Asymmetric PNA-DNA Duplexes: Differential Stabilization of PNA-DNA Duplexes by Purines in the PNA Strand. *Biophys. J*. **2006,***90,* 1329–1337.

e Ratilainen, T.; Holmén, A.; Tuite, E.; Haaima, G.; Christensen, L.; Nielsen, P.; Nordén, B. Hybridization of Peptide Nucleic Acid. *Biochemistry*. **1998,***37,* 12331–12342.

f Totsingan, F. Synthesis and Applications of PNA and Modified PNA in Nanobiotechnology. Ph.D. Thesis, University of Parma, Parma, Italy, **2007**.

g Sforza, S.; Haaima, G.; Marchelli, R.; Nielsen, P. Chiral Peptide Nucleic Acids (PNAs): Helix Handedness and DNA Recognition. *Eur. J. Org. Chem*. **1999,** 197–204.

h Jasiński, M.; Miszkiewicz, J.; Feig, M.; Trylska, J. Thermal Stability of Peptide Nucleic Acid Complexes. *J. Phys. Chem. B*. **2019,***123*, 8168–8177.

### GBSA Benchmarking

**Figure 5:**
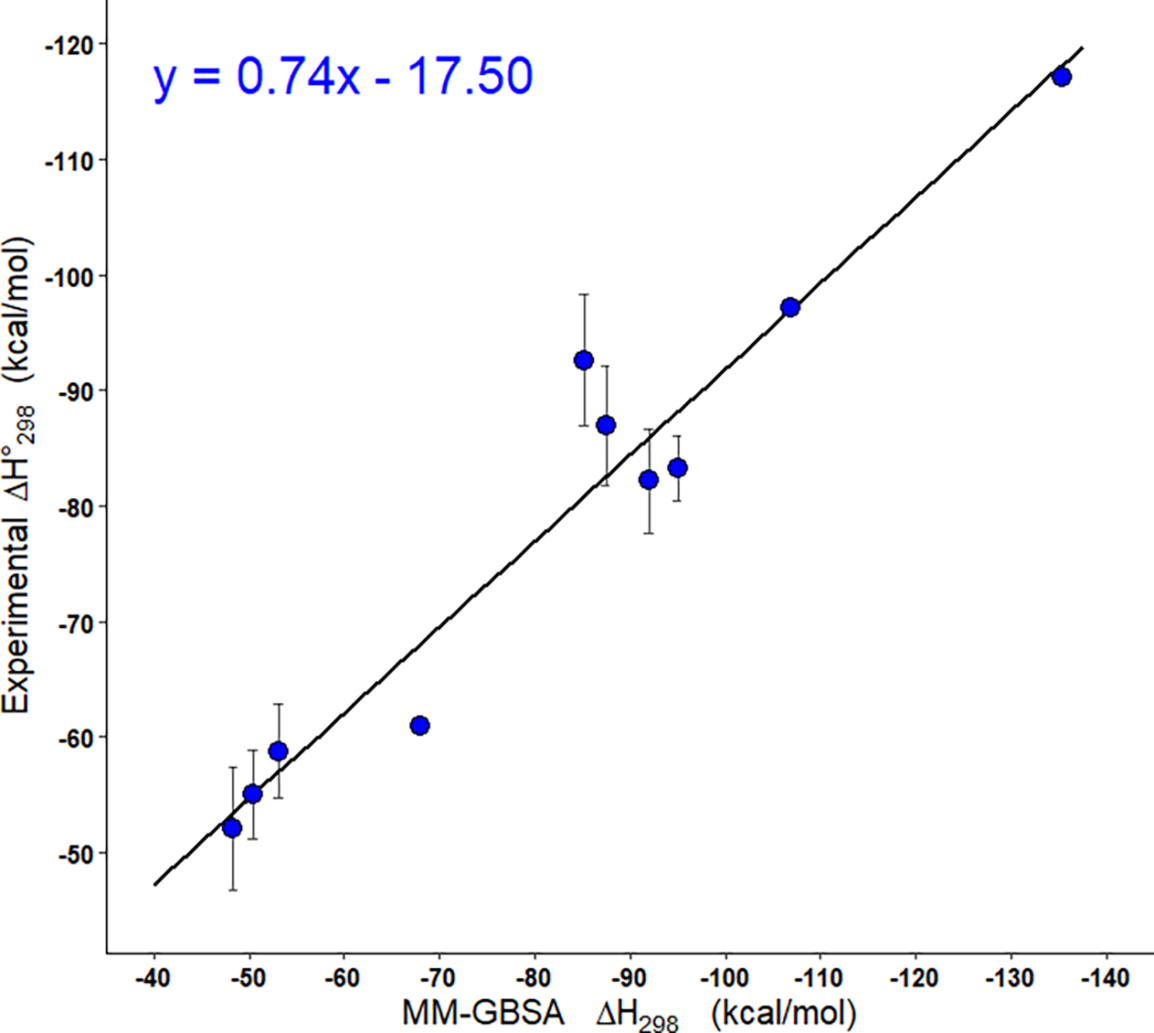
Correlation analysis for the enthalpy of binding of PNA homoduplexes from thermal melting experiments and from MM-GBSA analysis.

The entropies, enthalpies, and free energies of binding for the 10 PNA homoduplexes were obtained using MM-GBSA. Means and standard errors were obtained from triplicates. Linear regression of the enthalpies of binding from thermal melting experiments against MM-GBSA analysis produced an *R*^2^ of 0.93, indicating that they were well correlated. A high *R*^2^ would be anticipated given that there were few sequences of the same length, meaning a positive correlation requires only that the simulation and reality both report a higher enthalpy of binding for longer lengths, which is what was observed. The mean absolute difference, taken as the error of the prediction, between the enthalpy of binding from thermal melting and from MM-GBSA experiments was 7.87 kcal mol^–1^, and the mean relative error was 10.01%. The equation of the line between the thermal melting and MM-GBSA enthalpies (Figure 5) yielded a slope coefficient *a* = 0.74 and a shift coefficient *b* = −17.50. The large shift coefficient indicated a deviation between the thermal melting and MM-GBSA analysis. This was likely a result of a combination both of the assumptions made during simulation and of the small size of the data set, with only 10 sequences with thermal melting data being analyzed, causing the shift to be largely determined by only a few points.

Treatment of the binding enthalpies according to the equation of the line  reduced the relative and absolute errors of the estimates to 5.25% and 4.12 kcal mol^–1^, respectively.^2,3,14−18^

In contrast to the binding enthalpy, binding entropy and free energy were worse estimated, though they remained highly correlated with *R*^2^ of 0.84 and 0.86, respectively. The mean absolute and relative errors were 31.49 kcal mol^–1^ and 50.14% for *T*Δ*S* and 35.95 kcal mol^–1^ and 102.68% for the Gibbs free energy of binding. These errors are significantly higher than the error for the enthalpy and indicate that the configurational entropy as estimated by QH analysis does not accurately evaluate the entropy of binding from thermal melting experiments.

**Figure 6:**
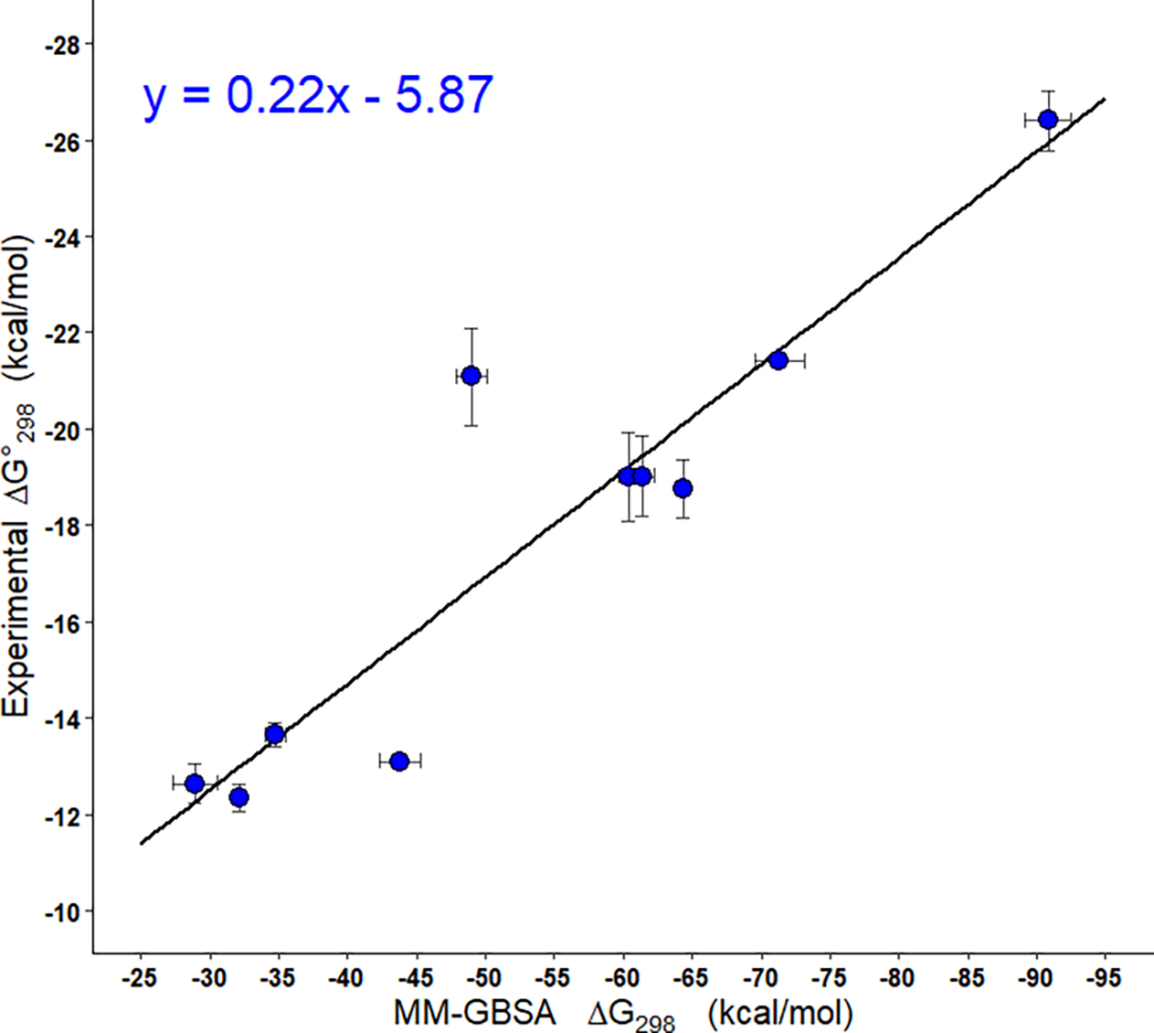
Correlation analysis for the Gibbs free energy of binding of PNA homoduplexes from thermal melting experiments and MM-GBSA.

**Figure 7:**
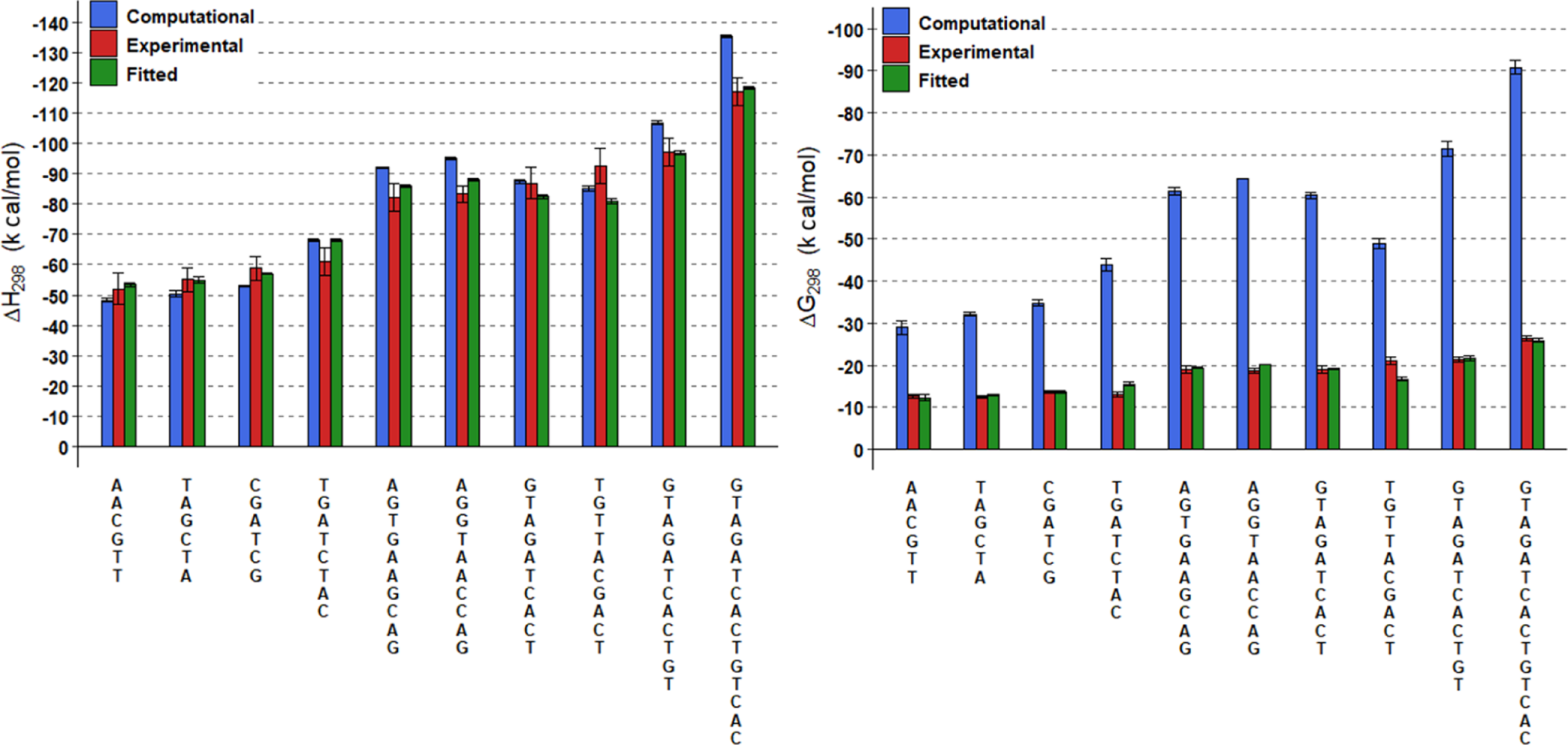
Enthalpies, on the left, and free energies, on the right, for the 10 PNA homoduplexes of the benchmarking set. Data from thermal melting experiments is in red. Data from MM-GBSA is in blue. Data after fitting according to *y* = *ax* + *b* is in green. Error bars indicate the standard errors. Standard errors for computational and fitted data are obtained from triplicate simulations.

Similarly to enthalpy, treatment of free energy according to the equation of the line,  (Figure 6) reduced the relative and absolute errors to 6.53% and 1.16 kcal mol^–1^, respectively. This demonstrates that significant correction can be achieved via linear regression, enabling the prediction of experimental binding energies from computer simulations even when there is quantitative disagreement (Figure 7).

### Nearest-Neighbor Modeling

To decompose binding energies into sequence parameters according to eqs 8 or 9, the coefficients in **ε** must converge. For that to be the case, the number of binding energies in **χ** must be large. The 10 PNA homoduplexes in Table 1 are not sufficient and so an additional 39 PNA homoduplexes were simulated (Table S3) and estimates of their binding enthalpies, entropies, and free energies were determined using MM-GBSA. A total of 49 PNA homoduplexes were used to produce the nearest-neighbor model. As a result, it is assumed that the errors described in the previous section are representative of the average error of the remaining 39 homoduplexes, which lack experimental data.

Benchmarking the subset of 10 sequences gave relative errors of 10.01 and 102.68% for the enthalpy and free energy of binding, respectively. A correction reduced these to 5.25 and 6.53%. This shows that thermal melting data can be accurately predicted using MM-GBSA without correction for enthalpy, though predictions of free energy require correction.

It is possible to develop a nearest-neighbor model with both corrected and uncorrected data. However, corrections according to the equations of the line, Δχ_exp_ = *a*Δχ_com_ + *b*, in Figures 5 and 6, impact the sequence parameters on the right-hand side of eq 7b in two ways
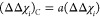
10a
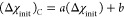
10b

where the subscript *C* indicates a term derived from solving the multiple regression problem using binding energies or entropies, which have been corrected according to Δχ_exp_ = *a*Δχ_com_ + *b*. The impact of the correction on the nearest-neighbor terms is not trivial. First, by scaling the stacked base pair energies according to the slope coefficient *a* (eq 10a), the equality ΔΔ*G* = ΔΔ*H* – *T*ΔΔ*S* only holds when *a*_1_ΔΔ*G* = *a*_2_ΔΔ*H* – *a*_3_*T*ΔΔ*S*, where *a*_*i*_ is the slope coefficient for the *i*th state variable such as the enthalpy. This is unlikely to be the case since the source of error differs between enthalpy and entropy: for enthalpy, it arises from the evaluation of potential energy, whereas for entropy, it arises from the evaluation of the fluctuations in atomic coordinates. In addition to this, the shift coefficient *b* may be large compared to the actual helix initiation energy or entropy. The corrected helix initiation value on the left-hand side of eq 10b thus may largely be determined by a metric of computational error as opposed to any physically relevant quantity.

So long as these considerations are taken into account, the model can be highly predictive of real-world measurements. This was the case for previous research on MM-GBSA estimates of DNA and RNA.^13^

In this research, corrected MM-GBSA data were not used for fitting the nearest-neighbor model so as to maximize the likelihood that sequence parameters were meaningful. Since the binding entropy, and therefore free energy, quantitatively disagreed with thermal melting data, a nearest-neighbor model was generated solely for the binding enthalpy. This was possible since, for the binding enthalpy, the experimental error was approximately equal to a typical experimental error of 10%.^13^

MM-GBSA enthalpies for all 49 sequences (Table S3) were used to solve the generalized multiple linear regression problem in eqs 5c–7a. Nearest-neighbor stacking, initiation, and terminal GC enthalpies were thus determined (Table 2).

**Table 2:** Nearest-Neighbor Binding Enthalpies for PNA Homoduplexes^a^

	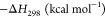	(kcal mol^–1^)
AA	8.65 ± 0.16	8.76 ± 0.31
AT	8.28 ± 0.32	
TA	9.34 ± 0.32	
AG	10.64 ± 0.27	9.72 ± 0.46
GA	8.91 ± 0.35	
AC	8.90 ± 0.19	
CA	10.31 ± 0.17	
GG	12.32 ± 0.19	10.91 ± 1.12
GC	8.69 ± 0.30	
CG	11.71 ± 0.34	
–Δ*H*_298_ (kcal mol^–1^)		
T.GG	1.62 ± 0.35	
Init	2.27 ± 0.46	

a ± indicates the standard error of the mean.

All parameters from Table 2 were significant to *p* < 10^–5^ in solving the multiple linear regression problem. Each stack was categorized according to the number of canonical Watson–Crick hydrogen bonds between them. These were six for GG, GC, and CG; five for AG, GA, AC, and CA; and four for AA, AT, and TA. On average, the stacking enthalpy became more negative as the number of Watson–Crick hydrogen bonds increased. This was in line with expectations since the hydrogen bonds stabilize the duplex.

The stacks GG and CG were more stabilizing relative to GC despite having the same number of hydrogen bonds. This difference could arise from the base stacking interaction, since base pairing only partly accounts for the energies of interaction between the strands. However, it may also reflect the sequence selection used in this study since bias can be introduced by particular stacks often coinciding or by a few sequences accounting for a large amount of the total occurrences of a stack.

Atypical for nucleic acid binding enthalpies is the negative helix initiation enthalpy. This enthalpy is typically positive for DNA and RNA duplexes.^48,49^ The explanation for it being stabilizing in PNA is due to PNA neutral backbone. In DNA and RNA, negative backbones repel, introducing a barrier to the association of the two strands. For PNA, however, this net repulsion does not occur, and so it is feasible that helix initiation is more favorable which would explain the negative helix initiation enthalpy. Similarly to other nucleic acid nearest-neighbor models, the terminal GC term is negative, indicating that the absence of terminal AT base pairs stabilizes the double helix.

The parameters in Table 2 can be used to estimate the binding enthalpies of PNA duplexes according to eq 7b (Table 3). Predicted binding enthalpies had a mean relative error of 8.74% when compared to thermal melting binding enthalpies. This was a decrease from the 10.01% error from the direct comparison of MM-GBSA to thermal melting data for the subset of 10 sequences. Since the nearest-neighbor model was based on a total of 49 sequences, the decrease in error showed that the accuracy of prediction increased by accounting for unrelated sequences.

**Table 3:** Experimental Binding Enthalpies Δ*H*_exp_^o^, Nearest-Neighbor Predicted Binding Enthalpies Δ*H*_NN_^o^, and Absolute  and Relative  Differences for 10 Homoduplexes

sequence	–Δ*H*_exp_^o^ (kcal mol^–1^)	–Δ*H*_NN_^o^ (kcal mol^–1^)	(kcal mol^–1^)	difference (%)
CGATCG	58.78 ± 4.09	53.40	5.38	9.15
AACGTT	52.05 ± 5.30	49.07	2.98	5.73
TAGCTA	55.08 ± 3.85	50.91	4.17	7.57
GTAGATCACT	86.92 ± 5.00	87.09	0.17	0.20
TGTTACGACT	92.60 ± 5.70	85.24	7.36	7.95
AGGTAACCAG	83.30 ± 2.80	92.28	8.98	10.78
AGTGAAGCAG	82.15 ± 4.56	89.95	7.80	9.49
TGATCTAC	60.90 ± 0.00	67.55	6.65	10.92
GTAGATCACTGT	97.10 ± 0.00	106.30	9.20	9.47
GTAGATCACTGTCAC	117.10 ± 0.00	136.03	18.93	16.17

This indicated that binding enthalpy could be readily predicted as a function of sequence parameters and that MM-GBSA could derive these sequence parameters. It is consequently possible that the nearest-neighbor model developed herein could aid in the design and structural understanding of PNA homoduplexes.

### Hydrogen Bond Analysis

Since the MM-GBSA nearest-neighbor predictions agree well with the available experimental data, it is possible to interpret them as having physical meaning. The first indication of this being the case is that, when the stacks are grouped according to the number of Watson–Crick hydrogen bonds involved, the binding enthalpy becomes, on average, more stable as the number of bonds increases.

Additional interpretations of physical meaning can be obtained through dynamics studies. The terminal GC term, T.GC, was investigated using hydrogen bond analysis. Watson–Crick hydrogen bonds of terminal and 3rd-position, classed as internal base pairs, were monitored. The number of Watson–Crick hydrogen bonds was normalized and these were expressed as percentages. These percentages were averaged over the simulation durations and for simulations involving sequences of the same length (Table 4).

**Table 4:** Mean Normalized Number of Hydrogen Bonds, between Zero and One, Expressed as a Percentage of Terminal and Internal AT and GC Base Pairs of Varying Sequence Lengths^a^

	% H-bonded	
length	AT	GC
	terminal	
6	70.4 ± 27.1	83.3 ± 4.3
8	74.4 ± 12.0	88.4 ± 2.9
10	84.7 ± 13.2	89.1 ± 2.1
12	79.3 ± 10.6	83.2 ± 7.1
14	80.3 ± 7.8	84.1 ± 6.5
16	68.4 ± 29.4	88.4 ± 1.7
18	67.6 ± 20.6	82.9 ± 3.9
	internal	
6	90.2 ± 2.4	95.7 ± 0.3
8	90.9 ± 0.5	94.0 ± 1.5
10	91.7 ± 0.9	95.3 ± 0.5
12	89.9 ± 1.6	96.0 ± 0.9
14	91.6 ± 0.7	96.0 ± 0.7
16	91.1 ± 0.4	95.0 ± 0.9
18	89.6 ± 2.4	95.8 ± 0.4

a Errors indicate standard deviations.

The percentage of unbroken bonds, from Table 4, is independent of sequence length and always, on average, higher for internal than terminal base pairs. Terminal AT hydrogen bonds were broken more often than terminal GC hydrogen bonds, though both spent most of their time in the bound state. Additionally, the standard deviation in the percentage of unbroken bonds was greater, most often by around 5-fold but in one case over 15-fold, for terminal AT than GC base pairs. This indicated that the terminal AT hydrogen bonding behavior was characterized by fluctuations between bound and melted states.

**Figure 8:**
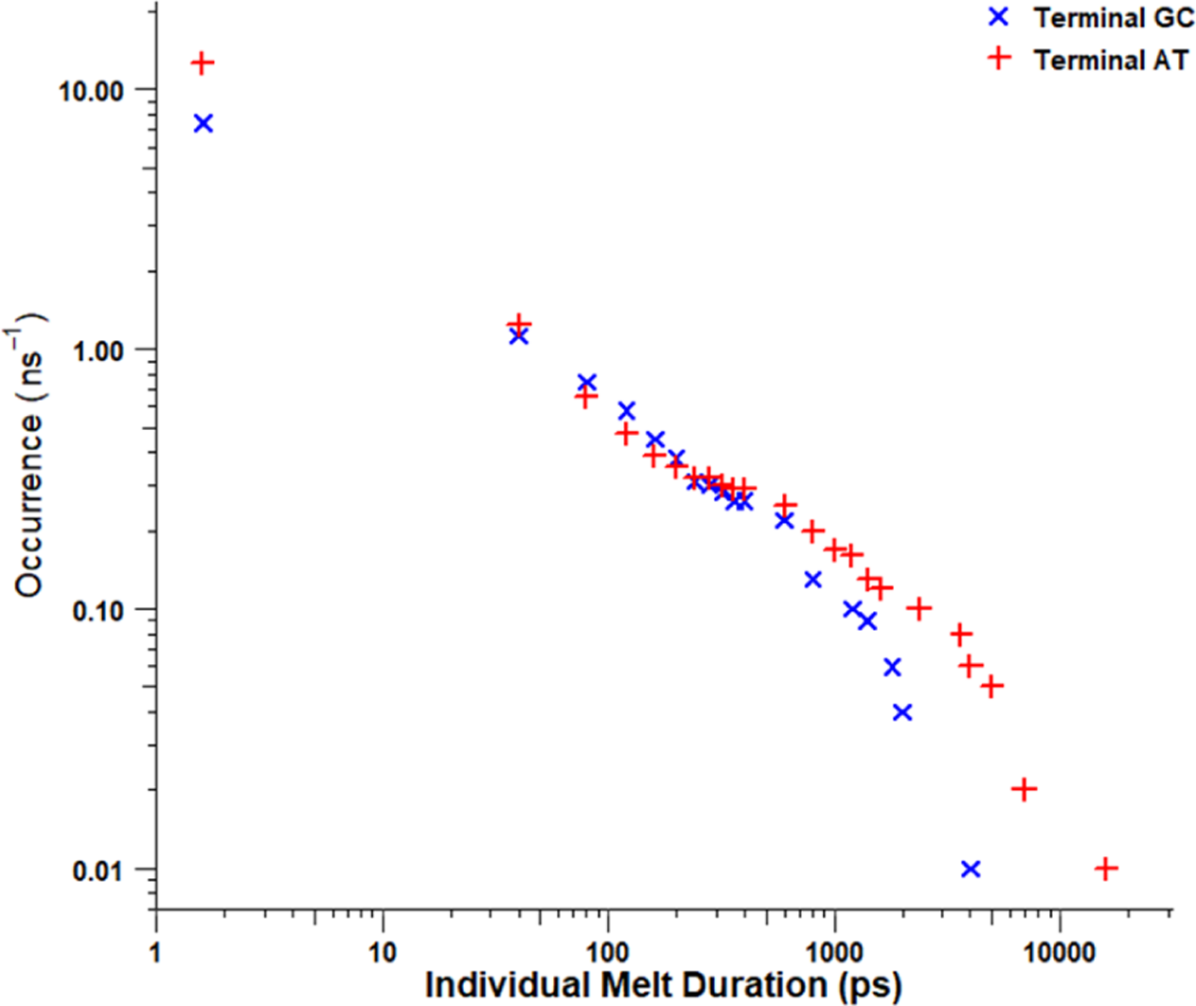
Occurrences per nanosecond of an individual melting event of at least a duration of picoseconds initiating for terminal GC base pairs, in blue, and terminal AT base pairs, in red.

To determine the nature of these fluctuations, the duration of the melting events was plotted against the occurrence, per nanosecond, of a melting event of that duration being initiated (Figure 8). A melting event was defined as an uninterrupted period of time with at least one hydrogen bond broken.

From Figure 8, it was observed that, for both AT and GC terminal base pairs, melting events of a duration less than one nanosecond were similarly common. By contrast, melting events longer than this were more common for terminal AT than GC pairs. This indicated that the large deviations in the number of unbroken bonds for the terminal AT pairs were likely a consequence of long, persistent melting events as opposed to short-lived ones. Since long melting events of this manner may destabilize the duplex and decrease its binding enthalpy, this may partly explain why the T.GC enthalpy, which is present only in the absence of AT termini, is negative.

In addition, the total melting time, obtained by aggregating all individual melting events during a run, was obtained. Total melting times for terminal (Figure S2) and internal (Figure S3) AT and GC base pairs were then plotted against the occurrence of a run with at least that much melting time out of all runs.

Consequently, the behavior observed in the hydrogen bonding analysis can corroborate the predictions of the nearest-neighbor model. This supports the hypothesis that an MM-GBSA nearest neighbor model contains meaningful quantities that relate energy with structure.

## Conclusions

Parameters for calculating the binding enthalpies of PNA duplexes as a function of their sequence have been developed. The difference between these predictions and PNA duplex binding enthalpies from thermal melting data was 8.74%. This was a reduction from an error of 10.01% from the direct comparison between MM-GBSA and thermal melting binding enthalpies. This suggested that the accuracy of the estimates improved when unrelated sequences were considered.

These results show that MM-GBSA can accurately decompose PNA binding enthalpies into parameters with structural meaning. This was demonstrated for the terminal GC term using a hydrogen bonding analysis, which showed that AT termini instabilities are characterized by persistent melting events.

Notably, the helix initiation enthalpy, which is positive for nearest-neighbor models of DNA and RNA,^48,49^ was negative for PNA. A plausible reason for this is that the backbone of PNA is neutrally charged, and hence association does not have to overcome the like-charge repulsion that occurs between the phosphoribose backbones of DNA or RNA. Despite its approximate nature, MM-GBSA was able to resolve binding enthalpies with accuracy comparable to experimental error. This research demonstrates the usefulness of MM-GBSA for predicting thermodynamic quantities in cases of limited experimental data, as would be expected for studies into novel nucleic acids.

## Supporting Information Available

Binding energies and entropies for all models; convergence of MM-GBSA outputs; rmsd traces of example models and replicas; and rmsd histograms of all replicas (PDF)
