# Supplementary material for: Modeling Peptide Nucleic Acid Binding Enthalpies Using MM-GBSA
Source: J Phys Chem B. 2022 Nov 14;126(46):9528–38. doi: 10.1021/acs.jpcb.2c05547 (PMC9706560; doi:10.1021/acs.jpcb.2c05547)
Supplement: Supplementary file 1 — jp2c05547_si_001.pdf [file jp2c05547_si_001.pdf]

Supporting Information

Modelling Peptide Nucleic Acid (PNA) Binding Enthalpies using MM-GBSA

Jack Goodman – University of the West of England, Bristol, United Kingdom, BS16 1QY

David Attwood – University of the West of England, Bristol, United Kingdom, BS16 1QY

Janice Kiely – University of the West of England, Bristol, United Kingdom, BS16 1QY

Richard Luxton – University of the West of England, Bristol, United Kingdom, BS16 1QY

Pablo Coladas Mato – GKN Aerospace, Bristol, United Kingdom, BS34 6FB

Table S1: Binding energies, enthalpies and free energies of GTAGATCACT from the literature. Uncategorised and sequences categorised according to the presence or absence of a lysine tag are included. $\pm$ indicates the standard error.

Category	$-\Delta G^o_{298}$ $(kcal\ mol^{-1})$	$-\Delta H^o_{298}$ $(kcal\ mol^{-1})$	$-\Delta S^o_{298}$ $(cal\ K^{-1}mol^{-1})$
All Sequences	18.99 ± 0.87	86.92 ± 5.00	227.97 ± 14.48
No Lysine	18.85 ± 0.85	89.36 ± 2.73	236.15 ± 8.28
Lysine	19.70 ± 1.16	85.45 ± 6.59	221.42 ± 18.83

Table S2: Enthalpies of single-stranded PNAs from the single-trajectory approach H_{STA} and from simulations of free, unbound strands (H_{UB}). The enthalpy of a single strand is given by the sum of its potential and solvation energies $\langle E_{MM}\rangle + \langle G_{solv}\rangle$ as described in the main text. $\Delta H_{conf.} = H_{STA} - H_{UB}$ and refers to the energy associated with conformational energy of duplex formation.

Strand	$-H_{STA}$ $(kcal\ mol^{-1})$	$-H_{UB}$ $(kcal\ mol^{-1})$	$\Delta H_{conf.}$ $(kcal\ mol^{-1})$
CGATCG	657.43 ± 2.97	658.48 ± 1.62	1.05
GTAGATCACT	1027.47 ± 6.64	1038.52 ± 1.38	11.05
CATCTAGTGA	943.32 ± 6.86	951.88 ± 2.25	8.56

Table S3: MM-GBSA binding enthalpies, entropies and free energies for all simulated homoduplexes. Standard errors obtained from triplicates.

Sequence	$-\Delta G^{o}_{298}$ $(kcal\ mol^{-1})$		$-\Delta H^{o}_{298}$ $(kcal\ mol^{-1}))$		$-\Delta S^{o}_{298}$ $(cal\ K^{-1} mol^{-1})$	
**AACTAGATACAGTAATTA**	96.87	± 1.45	161.00	± 2.63	227.03	± 0.81
**AAGGCCTT**	47.62	± 0.86	72.98	± 0.37	94.87	± 1.99
**AATGGCAGTCGT**	69.54	± 0.49	108.36	± 0.67	141.00	± 1.26
**AGGATTCGCCTGCCAGTG**	125.67	± 1.30	173.80	± 2.10	175.52	± 1.70
**AGGTTCATGATTTG**	88.73	± 0.22	125.46	± 0.41	134.44	± 0.69
**AGTAGA**	34.02	± 0.74	49.99	± 0.17	51.08	± 10.0
**ATCTAATTAG**	58.88	± 0.30	85.28	± 0.29	98.88	± 0.69
**CAAGTTCAAGTC**	74.06	± 1.16	106.08	± 0.97	118.31	± 2.05
**CCAGCGGAAG**	69.93	± 0.98	97.89	± 0.55	104.15	± 1.76
**CGTAATGCCGTAGG**	96.10	± 1.06	134.36	± 2.44	139.62	± 2.31
**GCCGGC**	42.46	± 0.27	57.80	± 0.58	58.96	± 12.9
**GGAACTAGTTGACGGC**	108.71	± 0.63	152.37	± 0.51	157.98	± 1.59
**GTAGATCACT**	60.34	± 0.62	87.45	± 0.66	101.19	± 0.97
**GTCGAGTT**	46.84	± 0.61	68.60	± 0.16	82.83	± 1.88
**GTCGTCGTCGTCGTCGTC**	118.60	± 0.65	168.97	± 0.56	180.82	± 1.25
**TAGCGGCCATTATT**	92.43	± 0.52	129.39	± 0.26	135.20	± 1.35
**TAGTTGCAGATCCTAT**	102.62	± 0.96	144.36	± 1.08	151.52	± 2.08
**TCAGGTAACTTGCCGT**	108.21	± 1.81	150.012	± 2.47	151.71	± 3.48
**TGCAGTCC**	50.05	± 1.28	72.56	± 1.03	85.30	± 3.49
**TGCGGGATATAT**	77.87	± 1.03	109.42	± 0.19	116.68	± 2.81
**TGTTACGACT**	49.00	± 1.17	85.23	± 0.84	131.83	± 2.92
**CGAACGATA**	50.94	± 0.75	78.99	± 0.05	103.27	± 2.50
**ATTTATTTACGT**	65.30	± 0.73	101.84	± 0.35	132.69	± 2.51
**GCTTGCTTATT**	63.95	± 0.73	95.03	± 0.13	114.10	± 3.69
**TTACGGGATT**	63.68	± 7.13	92.16	± 0.44	105.04	± 4.94
**AGAGCGAGCGCGCTTTT**	105.22	± 1.04	157.66	± 0.88	186.88	± 0.59
**CAATCAGGATATGCCG**	103.24	± 0.64	150.92	± 0.17	170.85	± 1.91
**ATCGGCCGTGTATATCCGAT**	129.83	± 0.51	189.45	± 0.40	211.47	± 1.17
**TCGTATTACTA**	64.01	± 1.27	95.44	± 0.76	115.27	± 3.10
**CATATATGGCGGATTA**	101.06	± 0.90	147.51	± 0.39	166.71	± 1.69
**GTGCTGGTGGC**	74.16	± 0.87	105.21	± 0.82	113.98	± 4.26
**TGCAAGCTTACACA**	85.38	± 1.83	126.30	± 0.09	147.82	± 7.02
**CGATTCGAGGCCAGTACG**	122.22	± 0.33	174.14	± 0.67	185.38	± 0.71
**GCGGCATGTACGGC**	92.61	± 0.57	134.69	± 0.30	151.78	± 1.92
**TCATGCAGGCCGGCCGCG**	127.66	± 0.80	181.31	± 1.93	191.14	± 2.87
**TCGTATAGCTCATATT**	95.53	± 1.59	141.82	± 0.34	166.18	± 3.58
**GCGGCTAAC**	56.61	± 0.79	83.37	± 0.35	98.97	± 1.62
**CGATCG**	34.72	± 0.75	53.08	± 0.21	69.94	± 2.04
**AACGTT**	28.94	± 1.57	48.34	± 0.58	73.48	± 5.34
**TAGCTA**	32.13	± 0.49	50.40	± 0.97	69.62	± 2.14
**AGGTAACCAG**	64.33	± 0.03	94.98	± 0.53	112.32	± 1.94

**AGTGAAGCAG**	61.37	± 0.86	92.00	± 0.33	112.24	± 1.77
**TGATCTAC**	43.82	± 1.47	68.02	± 0.41	90.05	± 6.30
**GTAGATCACTGT**	71.33	± 1.78	106.82	± 0.69	129.15	± 1.20
**GTAGATCACTGTCAC**	90.83	± 1.67	135.41	± 0.47	160.28	± 1.17
**GTAGATCACTGTCACAG**	108.15	± 1.99	157.30	± 0.17	175.91	± 1.78
**GTAGATCACTGTCACAGAT**	117.26	± 0.46	173.53	± 0.65	200.04	± 5.23
**GGAAGCTT**	46.95	± 0.53	70.69	± 1.41	88.51	± 2.06
**AGCCGGC**	45.58	± 0.46	67.20	± 0.64	81.03	± 0.67

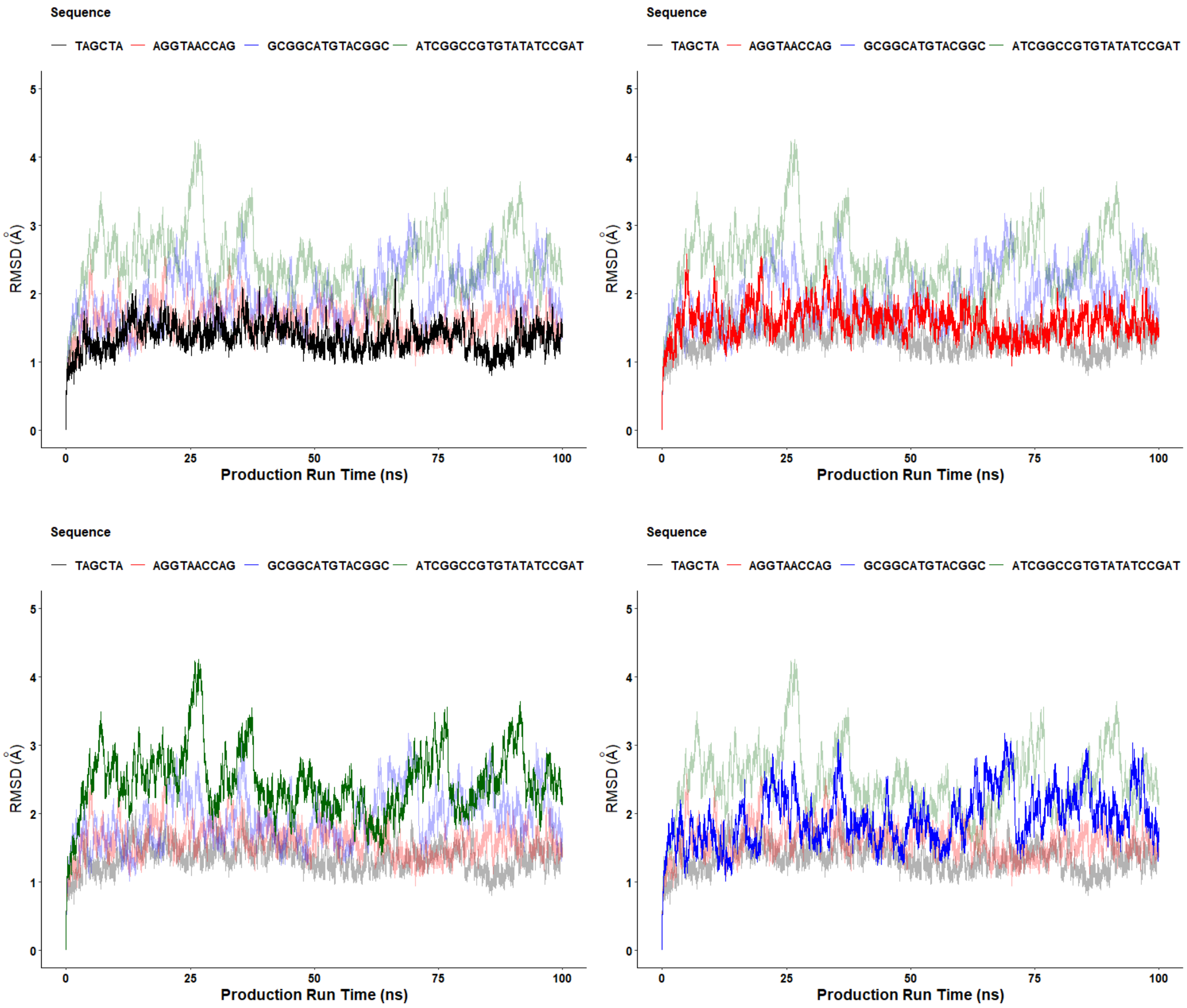

Figure S1: The root-mean-square atomic deviation (RMSD) in Angstrom of one replica each of four PNA homoduplexes of different length. Panels depict the same image with different traces highlighted for clarity.

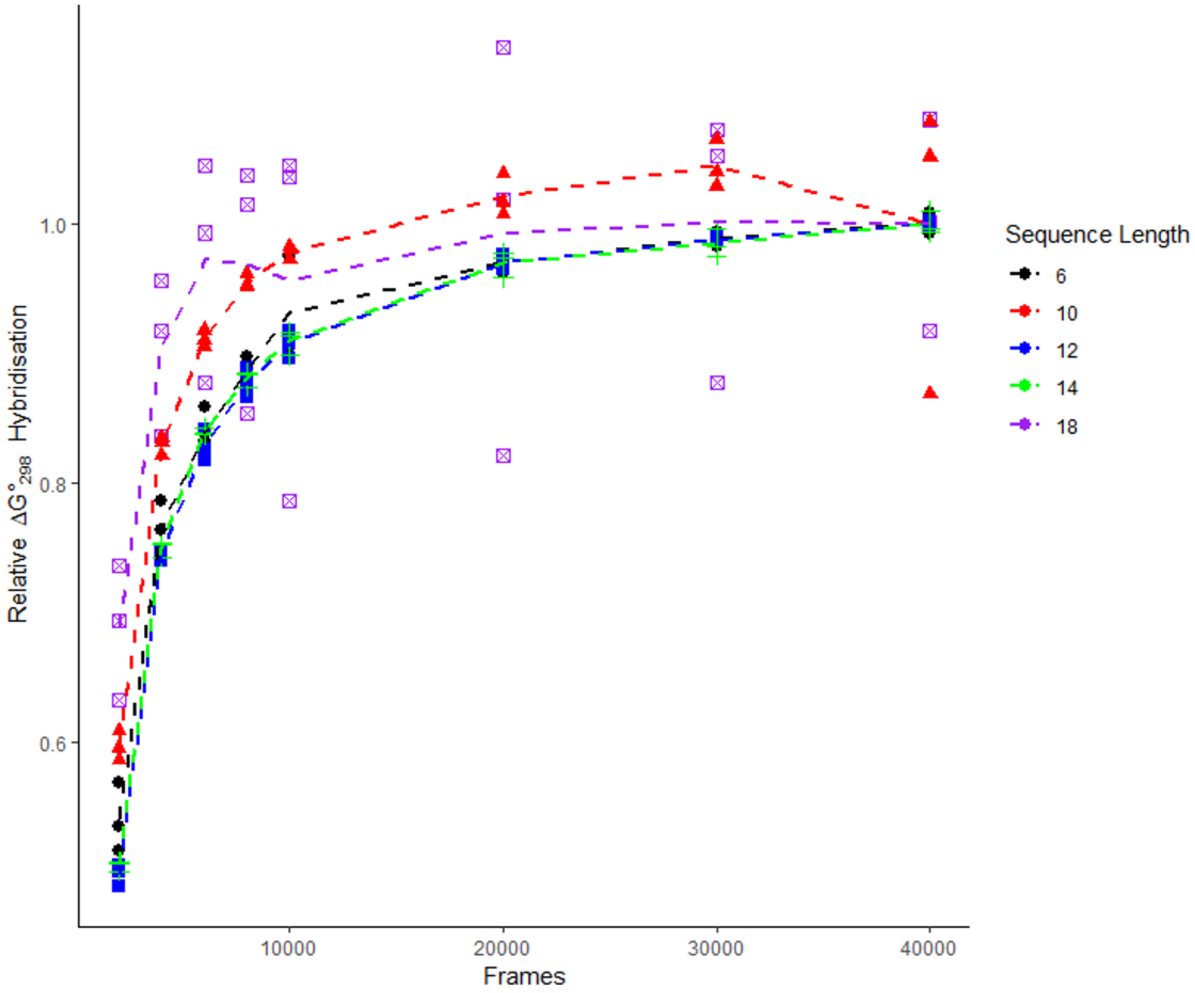

Figure S2: Convergence of MM-GBSA analysis of binding free energy for sequences of different length against the number of frames used. 40,000 frames corresponded to 80ns of simulation.

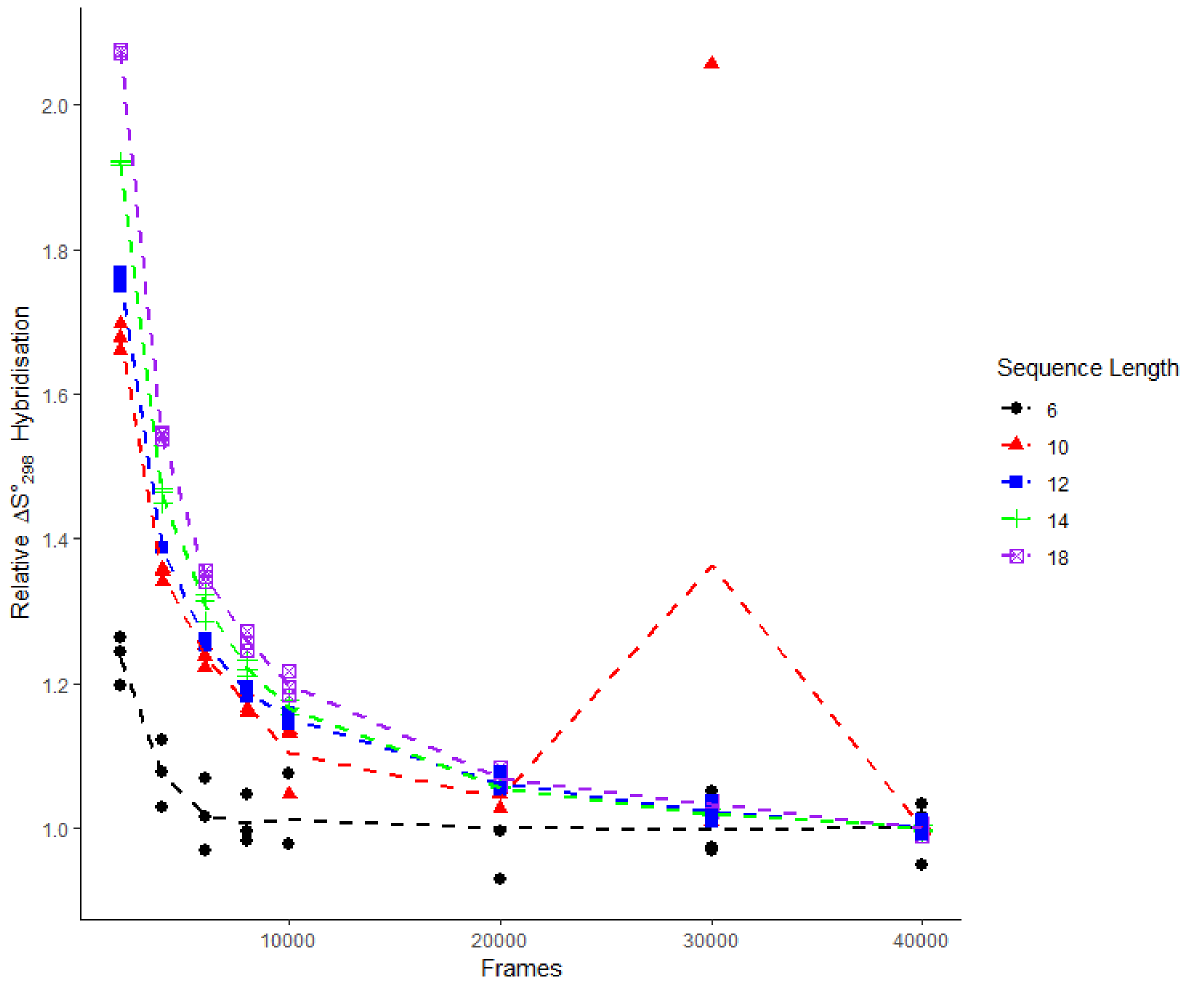

Figure S3: Convergence of QH analysis of binding entropy for sequences of different length against the number of frames used. 40,000 frames corresponded to 80ns of simulation.

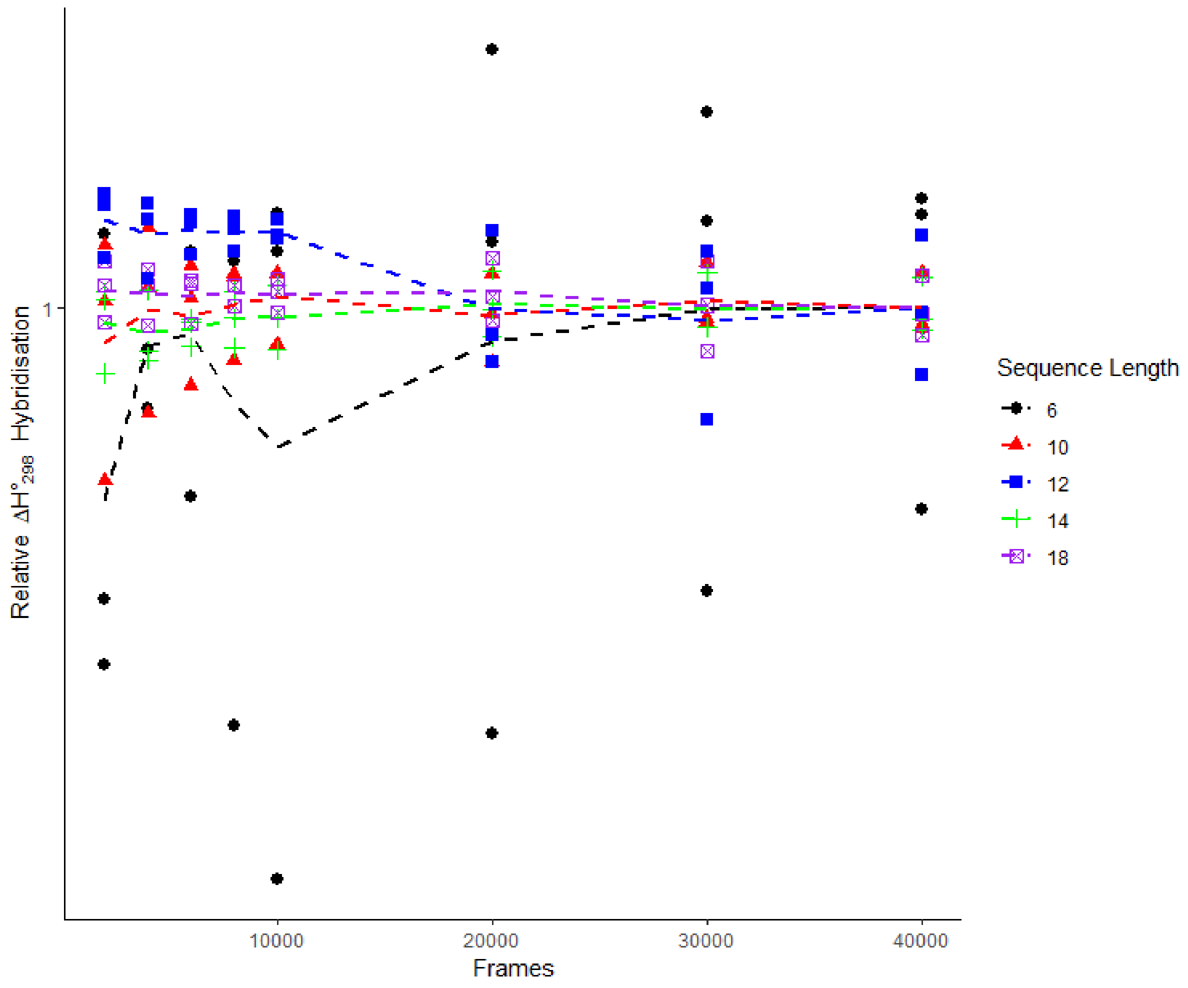

Figure S4: Convergence of MM-GBSA analysis of binding enthalpy for sequences of different length against the number of frames used. 40,000 frames corresponded to 80ns of simulation.

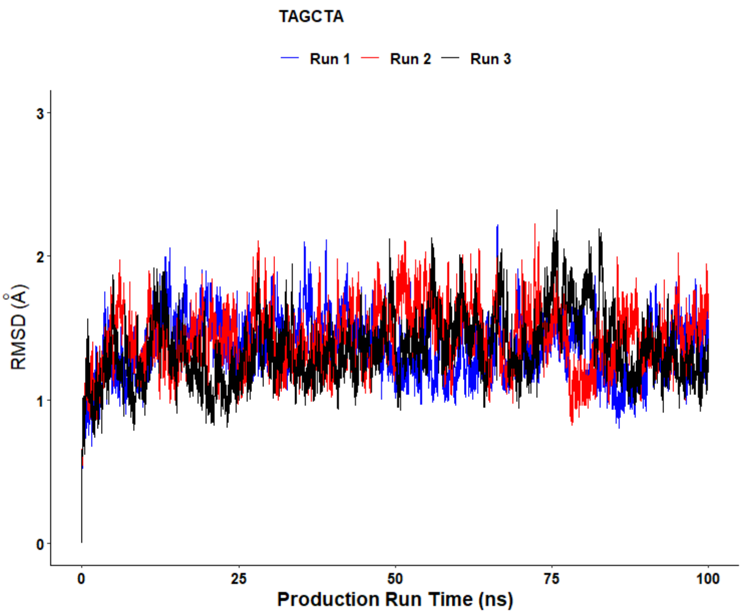
TAGCTA
Run 1
Run 2
Run 3
RMSD (Å)
0
1
2
3
0
25
50
75
100
Production Run Time (ns)

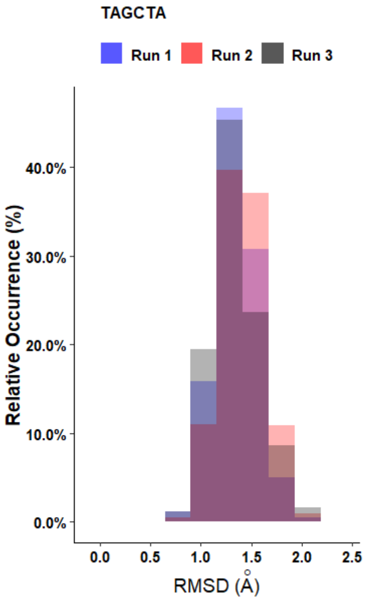
TAGCTA
Run 1
Run 2
Run 3
Relative Occurrence (%)
0.0%
10.0%
20.0%
30.0%
40.0%
0.0
0.5
1.0
1.5
2.0
2.5
RMSD (Å)

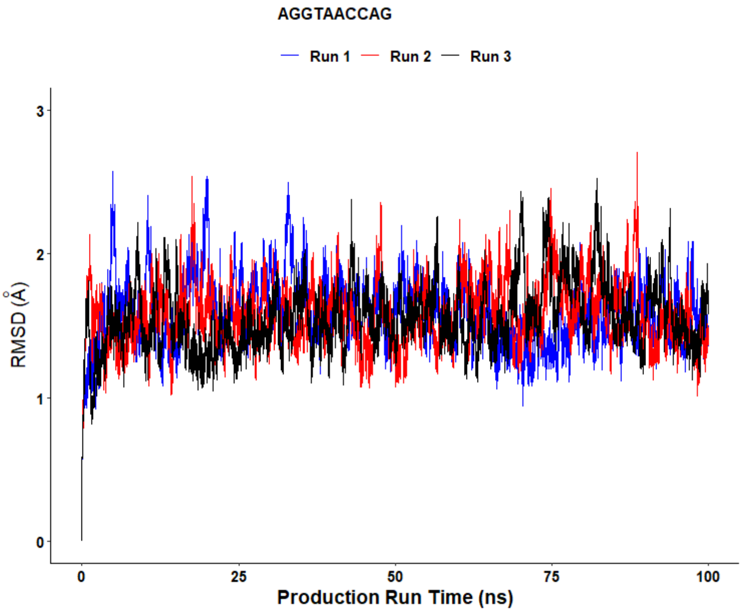
AGGTAACCAG
Run 1
Run 2
Run 3
RMSD (Å)
0
1
2
3
0
25
50
75
100
Production Run Time (ns)

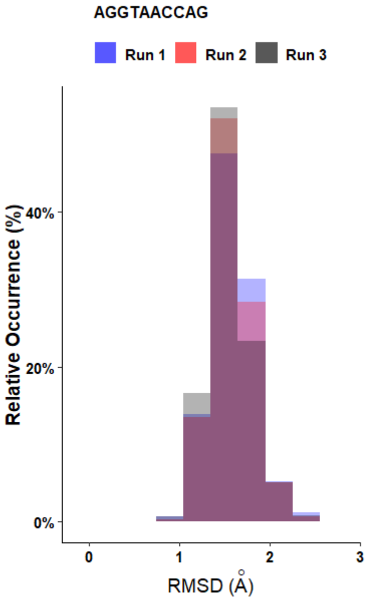
AGGTAACCAG
Run 1
Run 2
Run 3
Relative Occurrence (%)
0%
20%
40%
0
1
2
3
RMSD (Å)

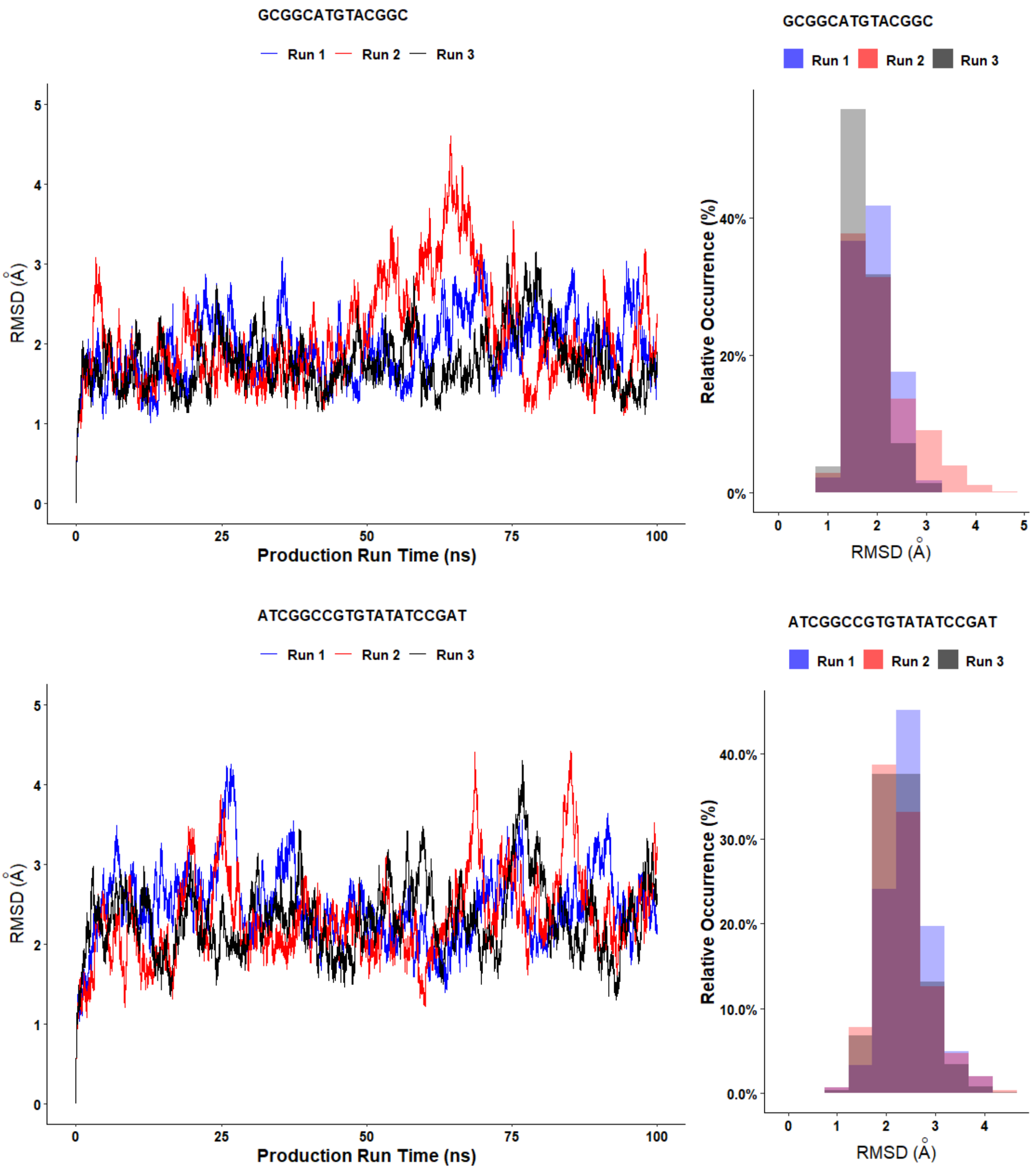

Figure S5: RMSD traces for the replicas of four example sequences with different lengths overlayed on one another down the left column against overlayed RMSD histograms of each replica for the four example sequences on the right. Sequence are, from top to bottom, TAGCTA, AGGTAACCAG, GCGGCATGTACGGC and ATCGGCCGTGTATATCCGAT. Sequence names are those of one strand and refer to a duplex whose opposing strand is its exact complement.

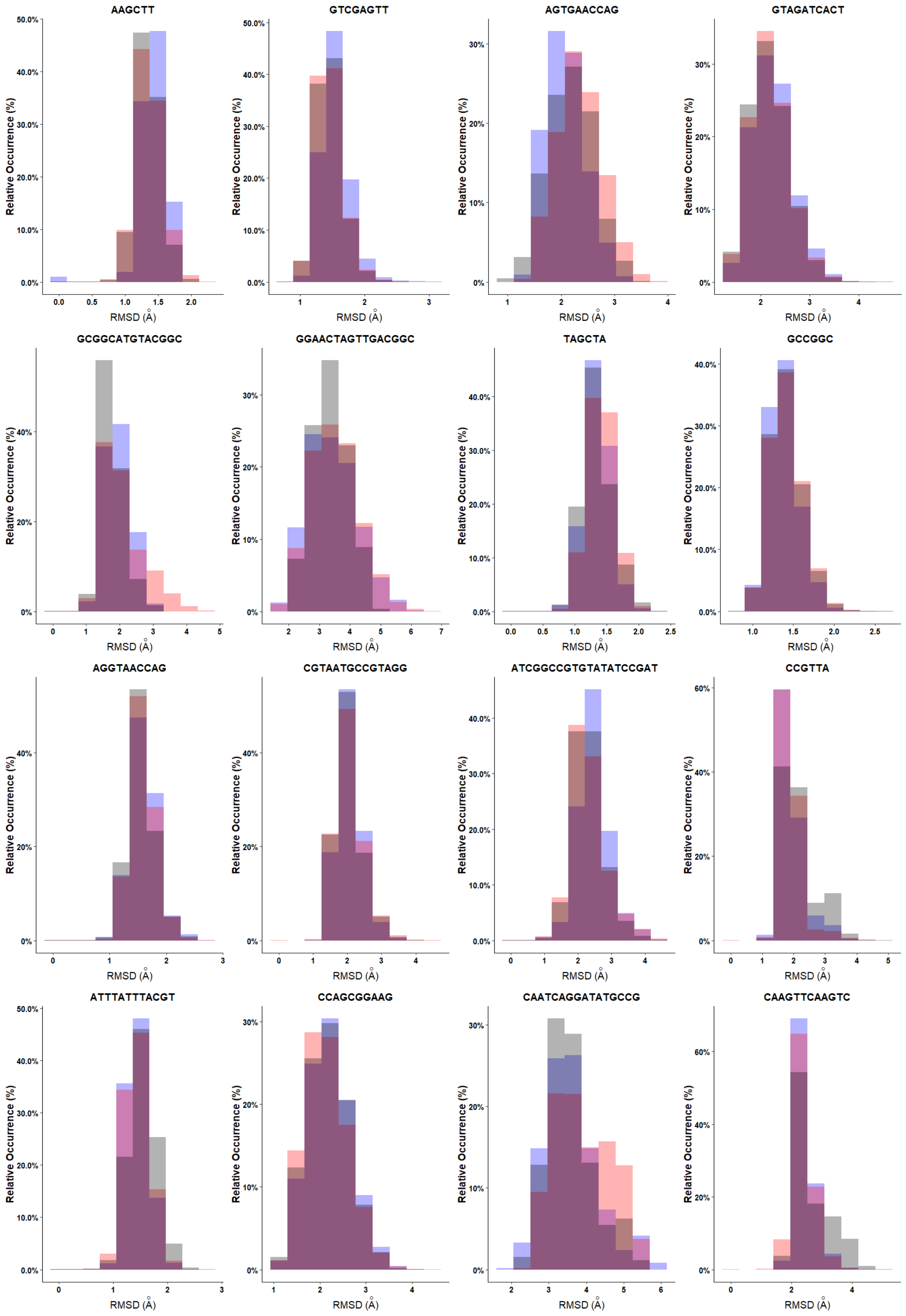
AAGCTT
GTCGAGTT
AGTGAACCAG
GTAGATCACT
GCGGCATGTACGGC
GGAACTAGTTGACGGC
TAGCTA
GCCGGC
AGGTAACCAG
CGTAATGCCGTAGG
ATCGGCCGTGTATATCCGAT
CCGTTA
ATTTATTTACGT
CCAGCGGAAG
CAATCAGGATATGCCG
CAAGTTCAAGTC
Relative Occurrence (%)
RMSD (Å)

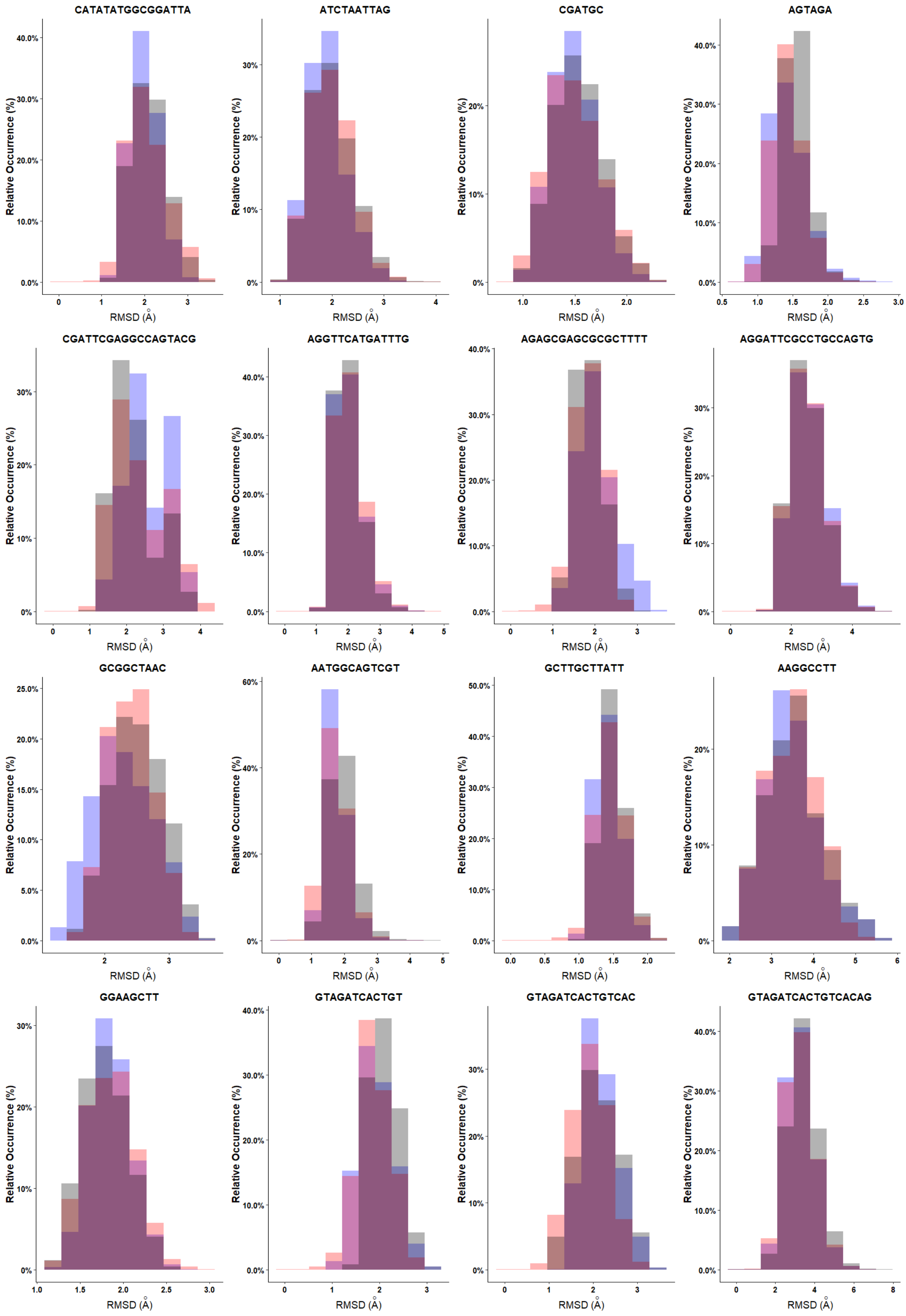
CATATATGGCGGATTA
ATCTAATTAG
CGATGC
AGTAGA
CGATTCGAGGCCAGTACG
AGGTTCATGATTTG
AGAGCGAGCGCGCTTTT
AGGATTCGCCTGCCAGTG
GCGGCTAAC
AATGGCAGTCGT
GCTTGCTTATT
AAGGCCTT
GGAAGCTT
GTAGATCACTGT
GTAGATCACTGTCAC
GTAGATCACTGTCACAG
Relative Occurrence (%)
RMSD (Å)

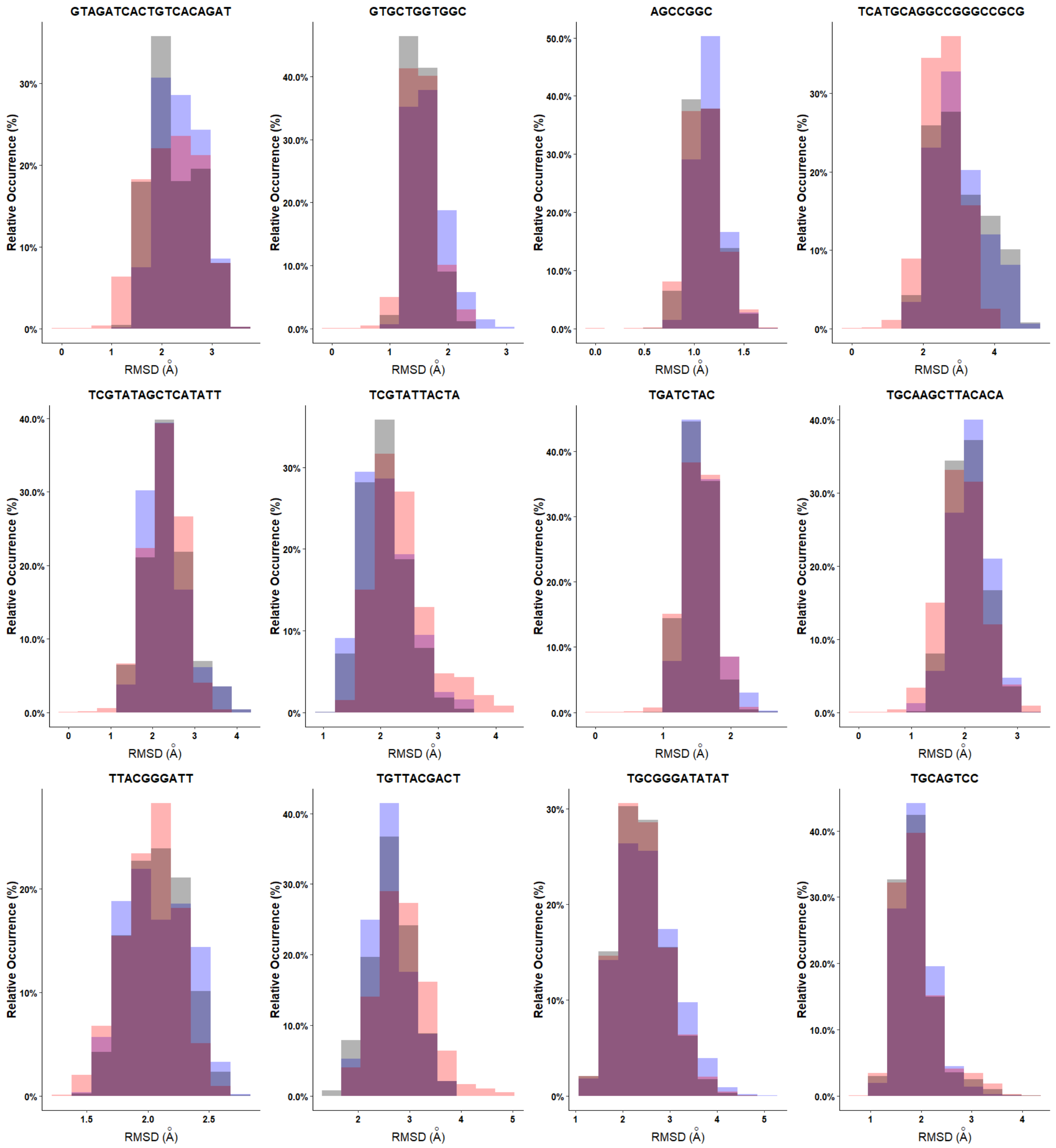

GTAGATCACTGTCACAGAT
GTGCTGGTGGC
AGCCGGC
TCATGCAGGCCGGGCCGCG
TCGTATAGCTCATATT
TCGTATTACTA
TGATCTAC
TGCAAGCTTACACA
TTACGGGATT
TGTTACGACT
TGCGGGATATAT
TGCAGTCC
Relative Occurrence (%)
RMSD (Å)

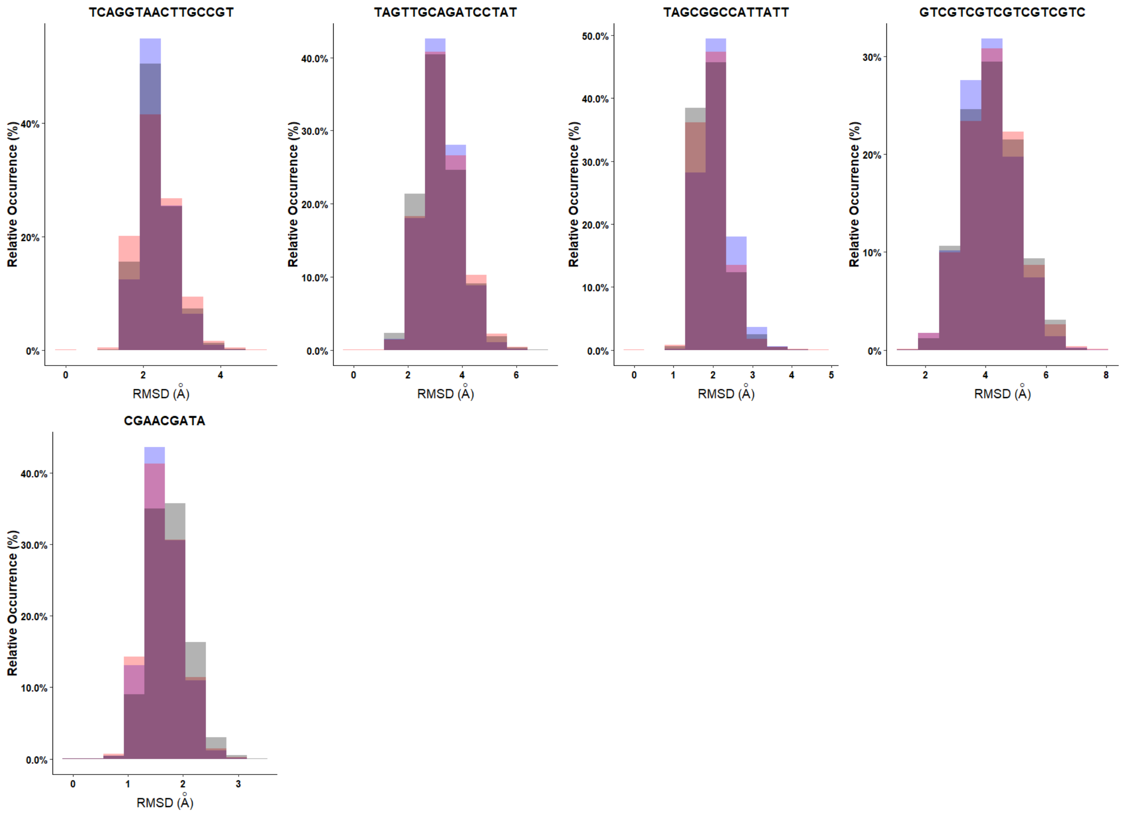

Figure S6: RMSD distribution histograms for each sequence overlayed with its replicas. Independent runs are coloured differently. Sequence names are those of one strand and refer to a duplex whose opposing strand is its exact complement.

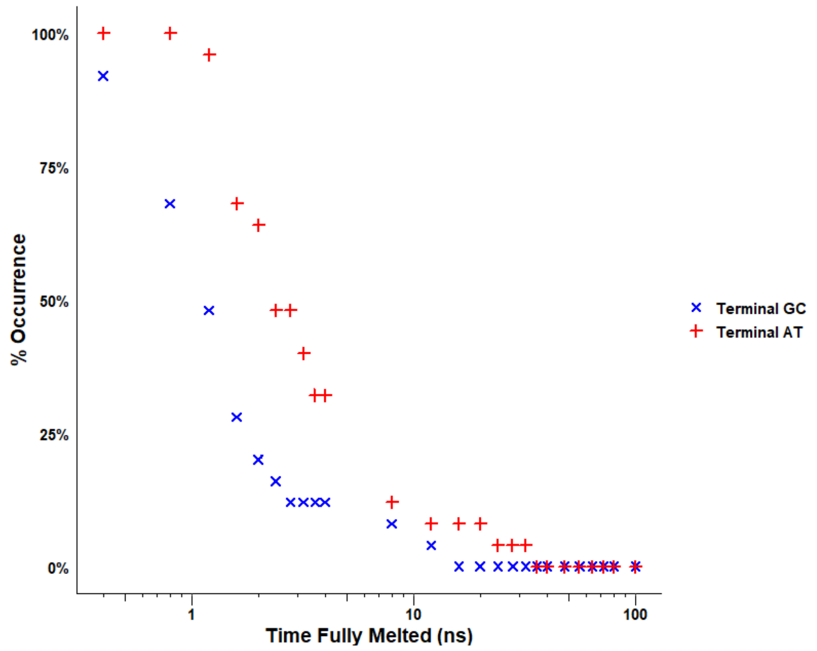

Figure S7: Occurrence of hydrogen-bonding trajectories for terminal base pairs against the total time they spent fully melted, wherein all hydrogen bonds are broken, for GC and AT base pairs.

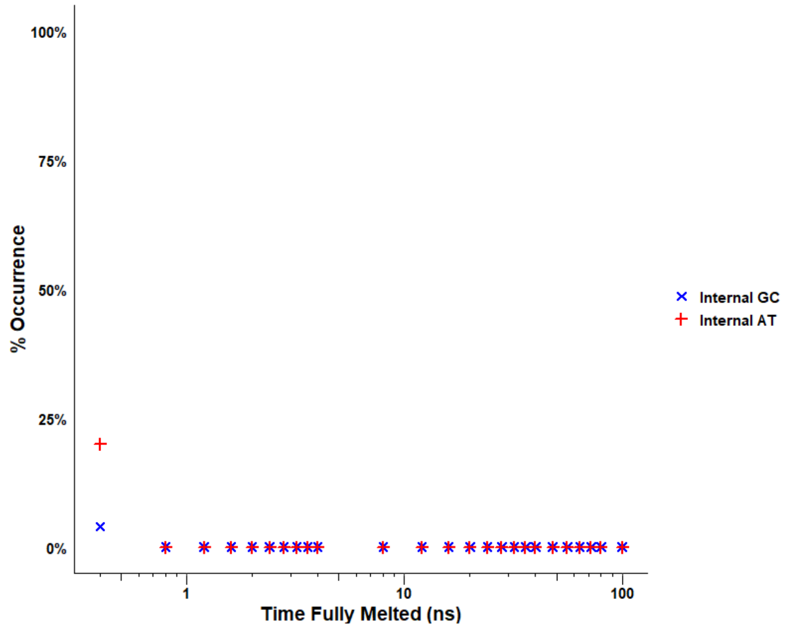

Figure S8: Occurrence of hydrogen-bonding trajectories for internal base pairs against the total time they spent fully melted, wherein all hydrogen bonds are broken, for GC and AT base pairs.
